# Synthesis and Biological Activity of Novel Oxazinyl Flavonoids as Antiviral and Anti-Phytopathogenic Fungus Agents

**DOI:** 10.3390/molecules27206875

**Published:** 2022-10-13

**Authors:** Yucong Ma, Lu Wang, Aidang Lu, Wei Xue

**Affiliations:** School of Chemical Engineering and Technology, Hebei University of Technology, Tianjin 300401, China

**Keywords:** natural product, oxazinyl flavonoids, anti-TMV activity, antifungal activity, mode of action, molecular docking

## Abstract

A series of oxazinyl flavonoids were synthesized on the basis of flavone. The structures of all target compounds were characterized by ^1^H NMR, ^13^C NMR, and HRMS. The effect of the different substituent on the N-position of oxazinyl flavonoids against tobacco mosaic virus (TMV) activities and plant pathogen activities was systematically investigated. In vivo anti-TMV activity showed that most of the compounds showed moderate-to-excellent antiviral activities against TMV at 500 μg/mL. Compounds **6b**, **6d**, **6j**–**6k**, and **6n**–**6q** showed better antiviral activities than ribavirin (a commercially available antiviral agent) and apigenin. In particular, compounds **6n** and **6p** even displayed slightly higher activities than ningnanmycin, which were expected to become new antiviral candidates. Antiviral mechanism research by molecular docking exhibited that compounds **6n** and **6p** could interact with TMV CP and inhibit virus assembly. Then, the antifungal activities of these compounds against six kinds of plant pathogenic fungi were tested, and the results showed that these oxazinyl flavonoids had broad-spectrum fungicidal activities. Compounds **6h** exhibited antifungal activity of up to 91% against *Physalospora piricola* and might become a candidate drug for new fungicides.

## 1. Introduction

In the process of plant growth, diseases caused by fungal and viral pathogens are often accompanied, causing significant economic losses to agricultural and horticultural production [1]. Although there are already widely used antiviral agents in the market, in recent years, with the rapid growth of the population and the rapid expansion of crop cultivation, the danger posed by plant virus diseases should still not be ignored. Tobacco mosaic virus (TMV) is one of the earliest and most extensively researched model viruses [2]. TMV, known as “plant cancer” [3], not only infects tobacco but also has a wide host range in more than 800 plant species from 65 families, such as peppers, tomatoes, eggplants, and potatoes [4]. It could greatly reduce crop yield and crop product quality [5,6]. Therefore, it is urgent to develop some new green pesticides.

In the design of new pesticides, natural products (NPs) have been widely selected to be lead compounds, with many inherent advantages, such as good biocompatibility, environmental friendliness, and novel modes of action [7,8,9,10]. In most heterocyclic compounds with excellent biological and pharmacological activities, the characteristics of “high efficiency and low toxicity” are often regarded as the key structures for the development of new pesticides [11,12]. Flavonoids are one of a large class of natural products that have long been recognized and that ubiquitously exist in plants [13]. So far, more than 8000 natural products have been classified as flavonoids. Although their structures are diverse, most of them are hydroxylated polyphenols with two or more aromatic rings and at least one aromatic hydroxyl group connected by heterocyclic pyrans [14]. According to their chemical structures, flavonoids are classified into chalcones, flavanones, flavanonols, flavones, flavonols, isoflavones, etc. [15]. Further studies describe that flavonoids have many pharmacological effects, which include antioxidant, analgesic, anti-inflammatory, anti-cancer, anti-microbial, anti-viral, and anti-malarial properties, among others [16,17,18,19]. Some natural flavonoids, such as quercetin, vitexin, fistulaflavonoids, and flavonoid glycosides, have been reported to exhibit anti-tobacco mosaic virus (anti-TMV) activities [20,21,22]. These results encouraged us to further modify flavonoids and apply them to protect crops. Oxazine compounds contain oxygen and nitrogen double heteroatoms, which can introduce various active pharmacophores on the N atom and have a wide range of biological activities [23]. Oxazinyl isoflavonoid compounds were also synthesized by Wang et al. [24] and Chen’s group [25], who exhibited potent anti-cancer activities.

**Figure 1:**
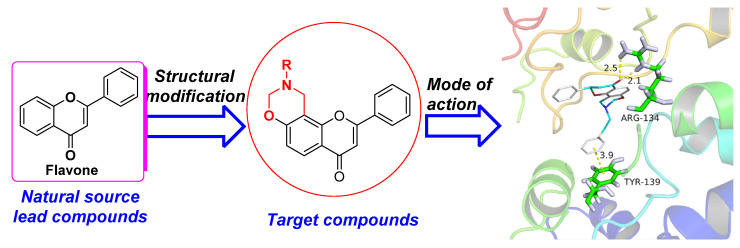
Design of flavonoids containing the oxazine structure.

In this paper, a series of flavone derivatives containing an 1,3-oxazine fragment were designed and synthesized on the basis of the natural product flavone (Figure 1). The structures of all target compounds were characterized by ^1^H NMR, ^13^C NMR, and HRMS. 7-Hydroxyflavone was selected as the mother nuclei, and the mother nucleus structure was modified by the integration of oxazinyl with the A rings of the mother nucleus structure to protect -OH on the 7-positions and the introduction of different substituents on N-positions of the oxazine ring to investigate the effect of the different groups on the activity. Then, antiviral and antifungal activities of these compounds and their structure–activity relationships were systematically evaluated. Antiviral mechanism research by molecular docking exhibited that oxazinyl flavonoid compounds could interact with TMV CP and inhibit virus assembly.

## 2. Results

### 2.1. Chemistry

**Scheme 1:**
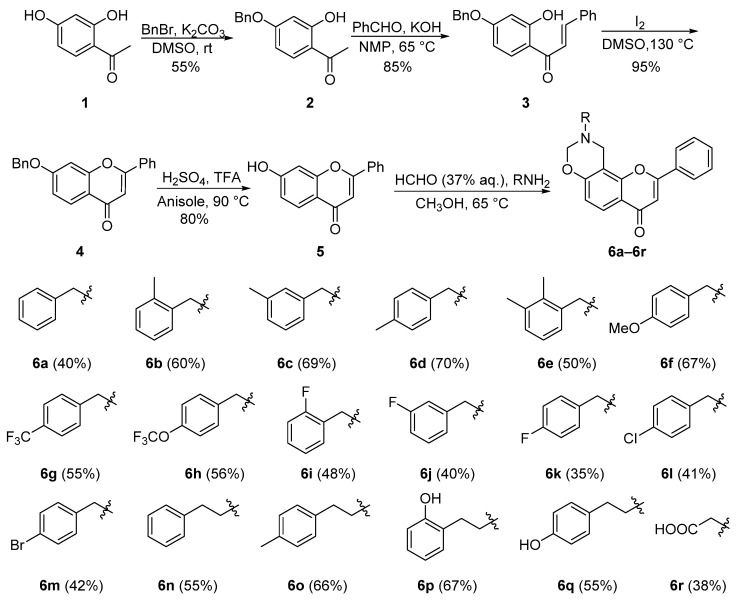
Synthesis of compounds **6a**–**6r.**

The raw material 2,4-dihydroxyacetophenone was purchased from the merchant. As shown in Scheme 1, the target compounds oxazinyl flavonoids were obtained from 2,4-dihydroxyacetophenone. Following an established method [26], the essential intermediate **5** was obtained. Subsequently, treatment of compound 5 with a primary amine and formaldehyde in methanol via a Mannich reaction afforded compounds **6a**–**6r [25]**. 

### 2.2. Phytotoxic Activity

After phytotoxic activity tests, the flavonoids containing the oxazine structure were found to be harmless to plants at a concentration of 500 μg/mL. Detailed testing procedures can be seen in our previous report [27,28] and can be found in the Supplementary Materials.

### 2.3. Antiviral Activity

#### In Vivo Anti-TMV Activity

**Figure 2:**
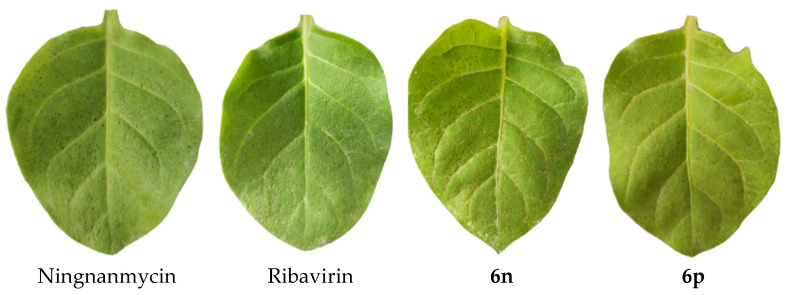
Inactivation activities of the compounds against TMV at 500 μg/mL.

The anti-TMV activities of oxazinyl flavonoids are listed in Table 1. The control drugs are the commercial ribavirin and ningnanmycin. Compounds **5** and **6a**–**6q** showed moderate to excellent anti-TMV activities; however, compound **6r** containing a carboxyethyl group had no inhibitory effect on TMV. All of the compounds containing phenylethyl groups exhibited higher anti-TMV activities than ribavirin. Compound **6p** presented the best anti-TMV activity at 500 μg/mL (inactivation activity, 63%; curative activity, 61%; protection activity, 62%), which was significantly higher than that of ribavirin (inactivation activity, 39%; curative activity, 38%; protection activity, 39%) and comparable to the control drug ningnamycin (inactivation activity, 60%; curative activity, 55%; protection activity, 57%) (Figure 2). According to the results in Table 1, compounds **6a**–**6f**, **6j**–**6k**, and **6n**–**6q** were selected for anti-TMV activity at 100 μg/mL. It was found that their inhibition rates of compounds **6a**–**6f**, **6j**–**6k**, and **6q** were about 10%, and that of compounds **6n**–**6o** were about 20%.

### 2.4. Mode of Action Studies

#### Docking Studies

To further study the mechanism of the interaction between oxazinyl flavonoids and TMV CP, we chose AutoDock Vina 1.1.2 for molecular docking [29]. The docking poses are ranked according to their docking sites, and the lowest binding energy of the macromolecule–ligand complex is considered the best. It can be proven that there is a H-bond interaction and strong binding affinity between oxazinyl flavonoids and TMV CP.

### 2.5. Fungicidal Activity

#### In Vitro Fungicidal Activity

Using chlorothalonil and carbendazim as controls, the inhibitory effects of compounds **5** and **6a**–**6r** on six common agricultural pathogens at 50 μg/mL were studied by fungicidal growth rate assay [29,30]. As shown in Table 2, most of the synthesized target compounds showed moderate-to-high inhibitory activities against the six plant pathogens in the in vitro test at a concentration of 50 μg/mL. The fungicidal activities of almost all the oxazinyl flavonoids **6a**–**6r** were higher than that of the natural product flavone. Compounds **6a**, **6c**, **6f**–**6h**, **6k**–**6m**, and **6r** showed inhibition rates higher than 50% against *Physalospora piricola*, and most compounds showed inhibition rates higher than 50% against *Sclerotinia sclerotiorum*. The inhibition rates of compounds **6a** and **6h** against *Physalospora piricola* reached 80% and 91%, respectively.

## 3. Discussion

### 3.1. Synthesis

According to the reported method [26], the 4-position hydroxyl group of 2,4-dihydroxyacetophenone was smoothly protected with benzyl bromide to obtain compound **2** and then treated with benzaldehyde in basic NMP by the Cleasen–Schmidt condensation to gain chalcone derivative **3**. Compound **3** converted to 7-benzyloxy flavonoid (**4**) by being oxidatively cyclized under the catalysis of iodine [31], which was then used to cleave the benzyl group in the mixed acid composed of TFA (trifluoroacetic acid) and concentrated sulfuric acid to yield **5**. According to the method reported in the literature [26], when compound **2** was used as raw material to prepare compound **3**, methanol was used as solvent, and the reaction was carried out at 25 °C for 15 h, wherein the actual yield was only 55%. In order to improve the yield, the reaction temperature was raised to 65 °C. Surprisingly, the yield was increased to 85%, and the reaction time was shortened to 3 h. In the process of preparing compound **4**, it was found that the amount of iodine was more than 0.1 equivalent, often with a by-product. The yield of compound **4** can be increased to 98% by reducing the amount of iodine to a 0.05 equivalent. Flavone derivatives **6a**–**6r** containing a 1,3-oxazine fragment were obtained by Mannich reaction of 7-hydroxyflavone, formaldehyde aqueous solution (37%), and primary amines with different substitutions.

### 3.2. Structure–Activity Relationship of the Antiviral Activity

The application of flavonoids in anti-virus has been reported, but there is limited research on plant viruses. Ten flavonoids were isolated from the seeds of *Aesculus chinensis* by Wei et al. and demonstrated some antiviral activities against respiratory syncytial virus (RSV), parainfluenza virus type 3 (PIV 3), and influenza virus type A (Flu A) [32]. Keum et al. [33] reported that natural products myricetin and scutellarein had strong inhibitory effects on the helicase of the SARS virus. Myricetin, quercetin, kaempferol, and flavanone were found to inhibit the SARS-CoV-2 nps13 unwinding activity at nanomolar concentrations [34]. Fortunately, as shown in Table 1, flavonoids also had certain antiviral activity against TMV, and most of them had better anti-TMV effects than the natural product apigenin and intermediate **5**. The integration of oxazinyl with the A rings of the mother nucleus structure to protect -OH on the 7-position showed increased activities, except **6r**, compared with apigenin, which may suggest that the increase in the steric hindrance of the flavone on 7,8-positions is favorable for activity. Comparing the antiviral activities of compounds **6a**–**6e**, the introduction of methyl substituent at the ortho and para positions of the benzene ring on N-positions of the oxazine ring to improve the anti-TMV activity, as well as the presence of methyl groups in the meta position of Ph, are unfavorable (**6d** > **6b** > **6a** ≈ **6e** ≈ **6c**). When the benzene ring of N-positions of oxazine ring contained MeO, CF_3_, CF_3_O, F, Cl, or Br, the rule of anti-TMV activity was not obvious. The result showed that the antiviral activity was influenced by the position of substituents on the benzene ring by comparing the antiviral effects of compounds **6a**–**6k**. When R was replaced by different phenethyl substituents on N-positions of the oxazine ring, the anti-TMV activity increased significantly (**6n** > **6a**, **6o** > **6d**, **6q** ≈ **6d**). In particular, compounds **6n** and **6p** were even slightly higher than ningnanmycin.

### 3.3. Study on the Mechanism of Anti-TMV Activity

#### Molecular Docking Study

**Figure 3:**
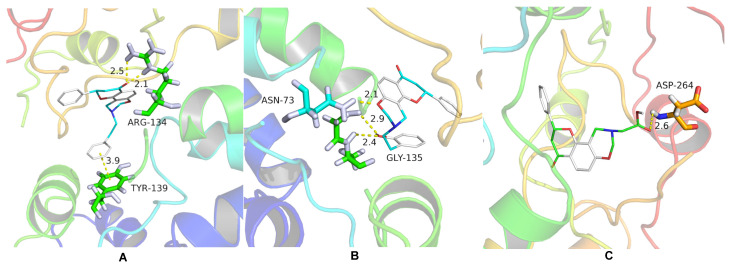
Molecule docking results of compounds **6n** (**A**), **6p** (**B**), and **6r** (**C**) with TMV CP.

Molecular docking studies were performed to explore the binding sites of oxazinyl flavonoids to TMV CP. The calculation procedures for molecular docking research were carried out according to the literature and are described in the Supporting Information [35]. Compounds **6n**, **6p**, and **6r** were chosen for molecular docking with TMV CP (PDB code 1EI7). The results showed that compound **6n** was laid into the TMV CP active pocket of ARG 134 and TYR 139 (Figure 3A). Compound **6n** formed two conventional hydrogen bonds with the active site of ARG 134 (2.1 Å and 2.5 Å), and the benzene ring also formed a π–π stacking interaction with TYR 139 (Figure 3A). Compound **6p** can provide O-H and O atoms to interact with amino acid residues and form intermolecular hydrogen bonds with the active sites of GLY 135 (2.4 Å and 2.9 Å) and ASN 73 (2.1 Å) (Figure 3B). Compared with compound **6r**, the carboxyl group could bond with ASP 264 (2.6 Å), as seen in Figure 3C. The molecular docking results indicated that these compounds interacted with CP through hydrogen bonding. In addition, the π–π stacking interaction of compound **6n** with TYR 139 strengthened the binding effect between the compound and TMV CP. The binding free energies of compounds **6n**, **6p**, and **6r** to TMV CP were −7.9 kcal/mol, −8.2 kcal/mol, and −7.3 kcal/mol, respectively. The lower the binding free energy, the higher the affinity between the receptor and the ligand [36]. The results showed that the introduction of different substituents into the N atom had a great impact on the interaction between the compounds and TMV CP. The stronger the affinity of the compound with TMV CP, the better the anti-TMV activity. It was also consistent with the activity test.

### 3.4. Structure–Activity Relationship of the Fungicidal Activity

Osonga et al. [37] reported that a new class of phosphorylated flavonoid compounds inhibited ultra-high inhibitory activities against *Listeria monocytogenes*, *Pseudomonas aeruginosa*, and *Aeromonas hydrophila*. 3-(Iso)quinolinyl-4-chromenone derivatives [38] were designed and synthesized to study their antifungal activities against *Colletotrichum orbiculare*, *Fusarium oxysporum*, *Sclerotinia sclerotiorum*, *Physalospora piricola*, *Valsa mali*, *Altenaria alternariae*, and *Botrytis cinerea*. The results showed that some compounds exhibited excellent in vitro antifungal activities against *Sclerotinia sclerotiorum*, *Valsa mali*, and *Botrytis cinerea*. Meenu et al. [39] found that the natural product cudraflavone C, as an acryl alcohol flavonoid, not only showed a significant inhibitory effect on *Staphylococcus aureus*, but also it was not easy to make it resistant. The activity test showed that the flavonoids synthesized in this paper also have broad-spectrum fungicidal activities. Compared with the natural product apigenin and intermediate **5**, as shown in Table 2, the introduction of oxazine structure enhanced these compounds’ activities against plant pathogens. The compounds containing electron-withdrawing groups on the benzene ring (**6f**, **6g**, **6j**, **6k**, **6l**, and **6m**) had higher inhibitory activities against *Physalospora piricola* than those containing electron-donating groups on the benzene ring (**6d**, **6e**, and **6q**). In addition, by comparing the fungicidal activities of compounds **6b**–**6d** and **6i**–**6k** against *Physalospora piricola*, it was found that when the benzene ring of the benzyl group was substituted by methyl, the order of activity was meta (**6c**) > para (**6d**) > ortho (**6b**), and when the benzene ring was substituted by fluorogen, the order of antifungal activity was ortho (**6i**) > meta (**6j**) > para (**6k**). The results manifested that the position of substituents on the benzene ring had an effect on the fungicidal activity. Furthermore, the fungicidal activity of compound **6e** with two methyl substituents on the benzene ring against *Sclerotinia sclerotiorum* was lower than that of compounds **6b** and **6c**, which indicated that the increase in substituents on the benzene ring was not conducive to the improvement of fungicidal activity.

## 4. Materials and Methods

### 4.1. General Procedures

#### 4.1.1. Instruments

The melting points of the products were determined on an X-4 binocular microscope (Gongyi Yuhua Instrument Co., Gongyi, China) and were not corrected. NMR spectra were acquired with a 400 MHz (100 MHz for ^13^C) instrument (Bruker, Billerica, MA, USA) at room temperature. Chemical shifts were measured relative to residual solvent peaks of DMSO-*d*_6_ as internal standards (^1^H: *δ* = 2.5 and 3.3 ppm; ^13^C: *δ* = 39.9 ppm). The following abbreviations are used to designate chemical shift multiplicities: s = singlet, d = doublet, dd = doublet of doublets, t = triplet, m = multiplet, and brs = broad singlet. HRMS data were recorded with a QFT-ESI instrument (Varian, Palo Alto, CA, USA). All reagents were of analytical reagent (AR) grade or chemically pure (CR). Compound **1** (AR) were purchased from Shanghai Bidepharm Co., Ltd. (Shanghai, China).

#### 4.1.2. Synthesis of Compounds **2**–**6**

1-[4-(Benzyloxy)-2-hydroxyphenyl]ethan-1-one (**2**). The K_2_CO_3_ (0.553 g, 4 mmol) and benzyl bromide (0.2 mL, 2 mmol) were added to the solution of 2,4-dihydroxyacetophenone (0.152 g, 1 mmol) in 2 mL of dimethyl sulfoxide (DMSO), and then the mixture was stirred at 25 °C overnight. After the reaction was completed, water was added to dilute and adjust the pH to 4–6, and the aqueous phase was extracted with ethyl acetate (10 × 5 mL) and the organic phases were combined, washed with saturated sodium chloride solution, dried with anhydrous sodium sulfate, and filtered. The crude product after evaporation was purified by column chromatography. White solid, 55% yield, m.p. 95–97 °C; ^1^H NMR (400 MHz, CDCl_3_) *δ* 12.73 (s, 1H), 7.64 (d, *J* = 8.5 Hz, 1H), 7.47–7.31 (m, 5H), 6.52 (d, *J* = 8.6 Hz, 2H), 5.10 (s, 2H), 2.56 (s, 3H); ^13^C NMR (101 MHz, CDCl_3_) *δ* 202.6, 165.2, 135.9, 132.4, 128.7, 128.3, 127.5, 114.1, 108.1, 101.9, 70.3, 26.3.

1-[4-(Benzyloxy)-2-hydroxyphenyl]-3-phenylprop-2-en-1-one (**3**). Benzaldehyde (0.212 g, 2 mmol) and potassium hydroxide (0.660 g, 10 mmol) were added to 5 mL of methanol, and then a mixed solution of **2** (0.242 g, 1 mmol) and NMP (*N*-methylpyrrolidone, 2 mL) was added dropwise. The mixture was stirred and refluxed at 65 °C until the raw materials disappeared. After the reaction, it was cooled to room temperature, diluted with water, adjusted to pH 2–4, and extracted with ethyl acetate (10 × 15 mL), and the organic phases were combined, washed with saturated sodium chloride solution, dried with anhydrous sodium sulfate, and filtered. The crude product after evaporation was purified by column chromatography. Yellow solid, 85% yield, m.p. 125–128 °C; ^1^H NMR (400 MHz, DMSO-*d_6_*) *δ* 13.39 (s, 1H), 8.30 (d, *J* = 9.0 Hz, 1H), 8.03 (d, *J* = 15.5 Hz, 1H), 7.92 (dt, *J* = 5.9 and 3.1 Hz, 2H), 7.83 (d, *J* = 15.5 Hz, 1H), 7.50–7.44 (m, 5H), 7.45–7.32 (m, 3H), 6.65 (dd, *J* = 8.9 and 2.5 Hz, 1H), 6.61 (d, *J* = 2.4 Hz, 1H), 5.22 (s, 2H); ^13^C NMR (101 MHz, DMSO-*d*_6_) *δ* 192.4, 166.1, 165.5, 144.7, 136.8, 135.0, 133.3, 131.3, 129.6, 129.4, 129.0, 128.6, 128.3, 121.7, 114.5, 108.5, 70.2.

7-(Benzyloxy)-2-phenyl-4*H*-chromen-4-one (**4**). To a solution of compound **3** (0.330 g, 1 mmol) in DMSO (5 mL), we added I_2_ (0.013 g, 0.05 mmol). The mixture was stirred and refluxed at 130 °C for 2 h. After this, the reaction was completed, diluted with water (5 mL), washed with the saturated sodium thiosulfate solution (3 × 10 mL) to remove I_2_, and then extracted with dichloromethane (10 × 20 mL). After filtration, the solvent was removed in vacuo to obtain compound **4**. White solid, 95% yield, m.p. 173–180 °C; ^1^H NMR (400 MHz, CDCl_3_) *δ* 8.16 (d, *J* = 8.6 Hz, 1H), 7.96–7.88 (m, 2H), 7.52 (d, *J* = 5.7 Hz, 3H), 7.43 (ddd, *J* = 22.6, 13.8 and 7.1 Hz, 5H), 7.07 (d, *J* = 9.7 Hz, 2H), 6.78 (s, 1H), 5.20 (s, 2H); ^13^C NMR (101 MHz, CDCl_3_) *δ* 177.9, 163.4, 163.2, 158.0, 135.8, 131.9, 131.5, 129.1, 128.8, 128.5, 127.6, 127.2, 126.3, 118.0, 115.0, 107.6, 101.6, 70.6.

7-Hydroxy-2-phenyl-4*H*-chromen-4-one (**5**). Anisole (0.5 mL) and concentrated sulfuric acid (0.2 mL) were added to the solution of **4** (0.328 g, 1 mmol) in trifluoroacetic acid (1.5 mL). The mixture was stirred and refluxed at 90 °C for 0.5 h. After the reaction, the solution was diluted with ice water (5 mL) and extracted with dichloromethane (3 × 10 mL); then, the organic phases were combined, washed with saturated sodium chloride solution, dried with anhydrous sodium sulfate, and filtered. The crude product after evaporation was purified by column chromatography. White solid, 80% yield, m.p. 160–166 °C; ^1^H NMR (400 MHz, DMSO-*d*_6_) *δ* 8.04 (d, *J* = 6.8 Hz, 2H), 7.84 (d, *J* = 8.6 Hz, 1H), 7.57 (s, 3H), 6.96–6.78 (m, 3H); ^13^C NMR (101 MHz, DMSO-*d*_6_) *δ* 176.8, 165.8, 162.1, 158.4, 132.0, 131.9, 129.6, 126.8, 126.6, 116.5, 115.5, 107.1, 102.9.

General procedure for **6a**–**6r**. Under the atmosphere of N_2_, **5** (0.238 g, 1 mmol) was dissolved in 40 mL of methanol, and different substituted primary amines (1.1 mmol) and formaldehyde aqueous solution (0.203 g, 2.5 mmol, 37%) were added. The mixture was stirred and refluxed at 65 °C for 36–60 h. After the disappearance of the raw material monitored by TLC (thin-layer chromatography), the solvent was removed in vacuo, and the crude product was purified by column chromatography.

9-Benzyl-2-phenyl-9,10-dihydrochromeno[8,7-*e*][1,3]oxazin-4(8*H*)-one (**6a**). White solid, 40% yield, m.p. 138–140 °C; ^1^H NMR (400 MHz, CDCl_3_) *δ* 8.04 (d, *J* = 8.8 Hz, 1H), 7.79 (d, *J* = 7.6 Hz, 2H), 7.50 (q, *J* = 6.1 Hz, 3H), 7.36 (dt, *J* = 21.2 and 6.7 Hz, 5H), 6.92 (d, *J* = 8.8 Hz, 1H), 6.77 (s, 1H), 4.96 (s, 2H), 4.34 (s, 2H), 3.96 (s, 2H); ^13^C NMR (101 MHz, CDCl_3_) *δ* 177.9, 162.4, 158.9, 154.7, 137.5, 131.9, 131.4, 129.1, 129.0, 128.6, 127.7, 126.0, 124.7, 117.5, 115.3, 107.8, 107.6, 82.4, 55.9, 45.4; HR-MS (ESI): calc’d for C_24_H_19_NO_3_ [M + H]^+^ 370.1438, found (ESI^+^) 370.1432.

9-(2-Methylbenzyl)-2-phenyl-9,10-dihydrochromeno[8,7-*e*][1,3]oxazin-4(8*H*)-one (**6b**). White solid, 60% yield, m.p. 124–128 °C; ^1^H NMR (400 MHz, CDCl_3_) *δ* 8.05 (d, *J* = 8.7 Hz, 1H), 7.80 (d, *J* = 7.4 Hz, 2H), 7.51 (d, *J* = 6.9 Hz, 3H), 7.30 (d, *J* = 6.9 Hz, 1H), 7.21 (q, *J* = 7.3 Hz, 3H), 6.93 (d, *J* = 8.8 Hz, 1H), 6.78 (s, 1H), 4.94 (s, 2H), 4.33 (s, 2H), 3.93 (s, 2H), 2.39 (s, 3H); ^13^C NMR (101 MHz, CDCl_3_) *δ* 178.0, 162.4, 159.0, 154.7, 137.7, 135.4, 131.9, 131.4, 130.6, 130.0, 129.1, 127.8, 126.0, 125.9, 124.7, 117.5, 115.3, 107.9, 107.6, 82.4, 53.8, 45.3, 19.1; HRMS (ESI) *m*/*z* calc’d for C_25_H_21_NO_3_ [M + H]^+^: 384.1585, found (ESI^+^) 384.1586.

9-(3-Methylbenzyl)-2-phenyl-9,10-dihydrochromeno[8,7-*e*][1,3]oxazin-4(8*H*)-one (**6c**). White solid, 69% yield, m.p. 128–134 °C; ^1^H NMR (400 MHz, CDCl_3_) *δ* 8.14–7.94 (m, 1H), 7.80 (d, *J* = 6.6 Hz, 2H), 7.50 (d, *J* = 7.3 Hz, 3H), 7.24 (s, 1H), 7.18 (d, *J* = 8.6 Hz, 2H), 7.13 (d, *J* = 7.3 Hz, 1H), 6.92 (d, *J* = 8.8 Hz, 1H), 6.77 (s, 1H), 4.97 (s, 2H), 4.33 (s, 2H), 3.92 (s, 2H), 2.35 (s, 3H); ^13^C NMR (101 MHz, CDCl_3_) *δ* 178.0, 162.5, 158.9, 154.8, 138.3, 137.4, 136.3, 131.9, 131.5, 130.3, 129.9, 129.1, 128.5, 126.0, 124.8, 121.2, 117.6, 115.3, 107.7, 82.4, 56.0, 45.4, 21.4; HRMS (ESI) *m*/*z* calc’d for C_25_H_21_NO_3_ [M + H]^+^: 384.1585, found (ESI^+^) 384.1585.

9–(4-Methylbenzyl)-2-phenyl-9,10-dihydrochromeno[8,7-*e*][1,3]oxazin-4(8*H*)-one (**6d**). White solid, 70% yield, m.p. 159–164 °C; ^1^H NMR (400 MHz, CDCl_3_) *δ* 8.03 (d, *J* = 8.8 Hz, 1H), 7.79 (d, *J* = 8.0 Hz, 2H), 7.50 (d, *J* = 7.4 Hz, 3H), 7.27 (d, *J* = 7.9 Hz, 2H), 7.17 (d, *J* = 7.6 Hz, 2H), 6.91 (d, *J* = 8.8 Hz, 1H), 6.77 (s, 1H), 4.95 (s, 2H), 4.32 (s, 2H), 3.91 (s, 2H), 2.36 (s, 3H); ^13^C NMR (101 MHz, CDCl_3_) *δ* 177.9, 162.5, 159.0, 154.8, 137.4, 134.5, 132.1, 131.4, 129.3, 129.1, 129.0, 126.0, 124.8, 117.6, 115.3, 107.9, 107.8, 82.4, 55.7, 45.3, 21.1; HRMS (ESI) *m*/*z* calc’d for C_25_H_21_NO_3_ [M + H]^+^: 384.1585, found (ESI^+^) 384.1587.

9-(2,3-Dimethylbenzyl)-2-phenyl-9,10-dihydrochromeno[8,7-*e*][1,3]oxazin-4(8*H*)-one (**6e**). White solid, 50.0% yield, m.p. 160–163 °C; ^1^H NMR (400 MHz, CDCl_3_) *δ* 8.05 (d, *J* = 8.8 Hz, 1H), 7.81 (d, *J* = 7.3 Hz, 2H), 7.51 (d, *J* = 6.8 Hz, 3H), 7.09 (dd, *J* = 17.3, 9.9 Hz, 3H), 6.93 (d, *J* = 8.9 Hz, 1H), 6.78 (s, 1H), 4.95 (s, 2H), 4.33 (s, 2H), 3.94 (s, 2H), 2.31 (d, *J* = 9.1 Hz, 6H); ^13^C NMR (101 MHz, CDCl_3_) *δ* 177.9, 162.4, 159.0, 154.7, 137.4, 136.3, 135.2, 131.9, 131.4, 129.6, 129.1, 128.2, 126.0, 125.3, 124.7, 117.5, 115.2, 107.8, 107.6, 82.3, 54.3, 45.2, 20.5, 15.0; HRMS (ESI) *m*/*z* calc’d for C_26_H_23_NO_3_ [M + H]^+^: 398.1751, found (ESI^+^) 398.1757.

9-(4-Methoxybenzyl)-2-phenyl-9,10-dihydrochromeno[8,7-*e*][1,3]oxazin-4(8*H*)-one (**6f**). White solid, 67% yield, m.p. 170–173 °C; ^1^H NMR (400 MHz, CDCl_3_) *δ* 8.04 (d, *J* = 8.8 Hz, 1H), 7.80 (d, *J* = 6.9 Hz, 2H), 7.51 (d, *J* = 6.9 Hz, 3H), 7.30 (d, *J* = 8.2 Hz, 2H), 6.91 (t, *J* = 7.7 Hz, 3H), 6.77 (s, 1H), 4.95 (s, 2H), 4.32 (s, 2H), 3.89 (s, 2H), 3.81 (s, 3H); ^13^C NMR (101 MHz, CDCl_3_) *δ* 178.0, 162.5, 159.2, 159.0, 154.8, 132.0, 131.5, 130.4, 129.5, 129.2, 126.1, 124.7, 117.6, 115.3, 114.0, 107.9, 82.2, 55.4, 55.3, 45.2; HRMS (ESI) *m*/*z* calc’d for C_25_H_21_NO_4_ [M + H]^+^: 400.1544, found (ESI^+^) 400.1548.

2-Phenyl-9-(4-(trifluoromethyl)benzyl)-9,10-dihydrochromeno[8,7-*e*][1,3]oxazin-4(8*H*)-one (**6g**). White solid, 55% yield, m.p. 158–162 °C; ^1^H NMR (400 MHz, CDCl_3_) *δ* 8.05 (d, *J* = 8.8 Hz, 1H), 7.77 (d, *J* = 7.9 Hz, 2H), 7.63 (d, *J* = 7.9 Hz, 2H), 7.51 (dd, *J* = 13.1 and 7.8 Hz, 5H), 6.93 (d, *J* = 8.8 Hz, 1H), 6.77 (s, 1H), 4.97 (s, 2H), 4.32 (s, 2H), 4.02 (s, 2H); ^13^C NMR (101 MHz, CDCl_3_) *δ* 177.9, 162.5, 158.7, 154.7, 141.6, 131.9, 131.6, 129.9, 129.2, 126.2, 126.0, 125.6 (*J*_C–-F_ = 4.0), 124.9, 122.8, 117.7, 115.3, 107.7, 107.6, 82.4, 55.5, 45.5; HRMS (ESI) *m*/*z* calc’d for C_25_H_18_F_3_NO_3_ [M + H]^+^: 438.1312, found (ESI^+^) 438.1318.

2-Phenyl-9-(4-(trifluoromethoxy)benzyl)-9,10-dihydrochromeno[8,7-*e*][1,3]oxazin-4(8*H*)-one (**6h**). White solid, 56% yield, m.p. 143–148 °C; ^1^H NMR (400 MHz, CDCl_3_) *δ* 8.04 (d, *J* = 8.8 Hz, 1H), 7.78 (d, *J* = 7.4 Hz, 2H), 7.50 (q, *J* = 7.1 and 6.6 Hz, 3H), 7.43 (d, *J* = 8.1 Hz, 2H), 7.22 (d, *J* = 8.0 Hz, 2H), 6.92 (d, *J* = 8.8 Hz, 1H), 6.77 (s, 1H), 4.96 (s, 2H), 4.31 (s, 2H), 3.96 (s, 2H); ^13^C NMR (101 MHz, CDCl_3_) *δ* 177.9, 162.5, 158.8, 154.7, 148.8, 136.3, 131.9, 131.5, 129.7 (*J*_C–F_ = 117.0), 126.0, 124.9, 121.8, 121.2, 119.3, 117.7, 115.3, 107.7, 107.6, 82.4, 55.2, 45.4; HRMS (ESI) *m*/*z* calc’d for C_25_H_18_F_3_NO_4_ [M + H]^+^: 454.1261, found (ESI^+^) 454.1269.

9-(2-Fluorobenzyl)-2-phenyl-9,10-dihydrochromeno[8,7-*e*][1,3]oxazin-4(8*H*)-one (**6i**). White solid, 48% yield, m.p. 128–131 °C; ^1^H NMR (400 MHz, CDCl_3_) *δ* 8.04 (d, *J* = 8.8 Hz, 1H), 7.81 (d, *J* = 7.9 Hz, 2H), 7.51 (d, *J* = 6.9 Hz, 3H), 7.44 (t, *J* = 7.8 Hz, 1H), 7.30 (dd, *J* = 9.2 and 3.2 Hz, 1H), 7.15 (t, *J* = 7.0 Hz, 1H), 7.07 (t, *J* = 9.1 Hz, 1H), 6.92 (d, *J* = 8.8 Hz, 1H), 6.78 (s, 1H), 4.99 (s, 2H), 4.36 (s, 2H), 4.02 (s, 2H); ^13^C NMR (101 MHz, CDCl_3_) *δ* 177.9, 162.6 (*J*_C–F_ = 246.0), 162.4, 158.8, 154.7, 131.9, 131.5, 131.3 (*J*_C–F_ = 4.0), 129.6 (*J*_C–F_ = 4.0), 129.1, 126.0, 124.8, 124.6, 124.1 (*J*_C–F_ = 3.0), 117.6, 115.6 (*J*_C–F_ = 22.0), 115.3, 107.7, 107.6, 82.5, 49.5, 45.5; HRMS (ESI) *m*/*z* calc’d for C_24_H_18_FNO_3_ [M + H]^+^: 388.1344, found (ESI^+^) 388.1347.

9-(3-Fluorobenzyl)-2-phenyl-9,10-dihydrochromeno[8,7-*e*][1,3]oxazin-4(8*H*)-one (**6j**). White solid, 40% yield, m.p. 132–136 °C; ^1^H NMR (400 MHz, CDCl_3_) *δ* 8.04 (d, *J* = 8.8 Hz, 1H), 7.79 (d, *J* = 7.3 Hz, 2H), 7.50 (d, *J* = 6.9 Hz, 3H), 7.32 (q, *J* = 7.3 Hz, 1H), 7.15 (t, *J* = 9.4 Hz, 2H), 7.01 (t, *J* = 8.5 Hz, 1H), 6.92 (d, *J* = 8.8 Hz, 1H), 6.77 (s, 1H), 4.96 (s, 2H), 4.32 (s, 2H), 3.96 (s, 2H); ^13^C NMR (101 MHz, CDCl_3_) *δ* 177.9, 163.1 (*J*_C–F_ = 244.9), 162.5, 158.8, 154.7, 140.3 (*J*_C–F_ = 7.0), 131.9, 131.5, 130.1 (*J*_C–F_ = 8.0), 129.2, 126.0, 125.6 (*J*_C–F_ = 3.0), 124.9, 124.4, 117.7, 115.8 (*J*_C–F_ = 20.0), 115.3, 114.8 (*J*_C–F_ = 21.0), 107.7, 82.5, 55.5, 45.5; HRMS (ESI) *m*/*z* calc’d for C_24_H_18_FNO_3_ [M + H]^+^: 388.1344, found (ESI^+^) 388.1347.

9-(4-Fluorobenzyl)-2-phenyl-9,10-dihydrochromeno[8,7-*e*][1,3]oxazin-4(8*H*)-one (**6k**). White solid, 35% yield, m.p. 140–143 °C; ^1^H NMR (400 MHz, CDCl_3_) *δ* 8.07 (d, *J* = 8.6 Hz, 1H), 7.82 (d, *J* = 7.9 Hz, 2H), 7.54 (d, *J* = 6.7 Hz, 3H), 7.45–7.34 (m, 2H), 7.09 (t, *J* = 8.2 Hz, 2H), 6.95 (d, *J* = 8.7 Hz, 1H), 6.86–6.76 (m, 1H), 4.98 (s, 2H), 4.35 (s, 2H), 3.96 (s, 2H); ^13^C NMR (101 MHz, CDCl_3_) *δ* 177.9, 162.6 (*J*_C–F_ = 245.0), 162.4, 158.8, 154.7, 133.2, 131.9, 131.5, 130.6 (*J*_C–F_ = 8.0), 129.1, 126.0 (*J*_C–F_ = 3.0), 124.8, 117.6, 115.4 (*J*_C–F_ = 21.0), 115.3, 107.7, 107.6, 82.2, 55.1, 45.3; HRMS (ESI) *m*/*z* calc’d for C_24_H_18_FNO_3_ [M + H]^+^: 388.1344, found (ESI^+^) 388.1347.

9-(4-Chlorobenzyl)-2-phenyl-9,10-dihydrochromeno[8,7-*e*][1,3]oxazin-4(8*H*)-one (**6l**). White solid, 41% yield, m.p. 171–180 °C; ^1^H NMR (400 MHz, CDCl_3_) *δ* 8.04 (d, *J* = 8.7 Hz, 1H), 7.78 (d, *J* = 5.5 Hz, 2H), 7.51 (d, *J* = 6.4 Hz, 3H), 7.33 (s, 4H), 6.91 (d, *J* = 8.8 Hz, 1H), 6.77 (s, 1H), 4.95 (s, 2H), 4.30 (s, 2H), 3.92 (s, 2H); ^13^C NMR (101 MHz, CDCl_3_) *δ* 177.8, 162.5, 158.8, 154.7, 136.1, 133.5, 132.0, 131.5, 130.3, 129.1, 128.8, 126.0, 124.9, 117.7, 115.3, 107.8, 107.7, 82.4, 55.3, 45.4; HRMS (ESI) *m*/*z* calc’d for C_24_H_18_ClNO_3_ [M + H]^+^: 404.1048 and 406.1018, found (ESI^+^) 404.1056 and 406.1025.

9-(4-Bromobenzyl)-2-phenyl-9,10-dihydrochromeno[8,7-*e*][1,3]oxazin-4(8*H*)-one (**6m**). White solid, 42% yield, m.p. 188–190 °C; ^1^H NMR (400 MHz, CDCl_3_) *δ* 8.04 (d, *J* = 8.8 Hz, 1H), 7.78 (d, *J* = 7.5 Hz, 2H), 7.59–7.42 (m, 5H), 7.27 (d, *J* = 9.8 Hz, 2H), 6.91 (d, *J* = 8.8 Hz, 1H), 6.76 (s, 1H), 4.95 (s, 2H), 4.30 (s, 2H), 3.91 (s, 2H); ^13^C NMR (101 MHz, CDCl_3_) *δ* 177.9, 162.5, 158.8, 154.7, 136.6, 131.9, 131.8, 131.5, 130.7, 129.2, 126.2, 124.9, 121.6, 117.7, 115.3, 107.8, 107.7, 82.4, 55.3, 45.4; HRMS (ESI) *m*/*z* calc’d for C_24_H_18_BrNO_3_ [M + H]^+^: 448.0543 and 450.0522, found (ESI^+^) 448.0549 and 450.0528.

9-Phenethyl-2-phenyl-9,10-dihydrochromeno[8,7-*e*][1,3]oxazin-4(8*H*)-one (**6n**). White solid, 55% yield, m.p. 135–139 °C; ^1^H NMR (400 MHz, CDCl_3_) *δ* 8.00 (d, *J* = 8.8 Hz, 1H), 7.89–7.75 (m, 2H), 7.54 (s, 3H), 7.27–7.17 (m, 5H), 6.87 (d, *J* = 8.8 Hz, 1H), 6.76 (s, 1H), 5.00 (s, 2H), 4.30 (s, 2H), 3.09 (t, *J* = 7.1 Hz, 2H), 2.93 (t, *J* = 7.0 Hz, 2H); ^13^C NMR (101 MHz, CDCl3) *δ* 178.0, 162.4, 158.9, 154.7, 137.5, 131.9, 131.5, 129.1, 128.6, 127.7, 126.0, 124.7, 117.6, 115.3, 107.8, 107.7, 82.4, 55.9, 45.4, 33.4; HRMS (ESI) *m*/*z* calc’d for C_25_H_21_NO_3_ [M + H]^+^: 384.1594, found (ESI^+^) 384.1597.

9-(4-Methylphenethyl)-2-phenyl-9,10-dihydrochromeno[8,7-*e*][1,3]oxazin-4(8*H*)-one (**6o**). White solid, 66% yield, m.p. 137–146 °C; ^1^H NMR (400 MHz, CDCl_3_) *δ* 8.00 (d, J = 8.8 Hz, 1H), 7.84 (d, *J* = 6.5 Hz, 2H), 7.54 (d, *J* = 4.7 Hz, 3H), 7.09 (q, *J* = 7.8 Hz, 4H), 6.87 (d, *J* = 8.8 Hz, 1H), 6.77 (s, 1H), 5.01 (s, 2H), 4.32 (s, 2H), 3.07 (t, *J* = 7.4 Hz, 2H), 2.89 (t, *J* = 7.3 Hz, 2H), 2.27 (s, 3H); ^13^C NMR (101 MHz, CDCl_3_) *δ* 178.0, 162.4, 159.0, 154.6, 136.4, 135.8, 132.0, 131.5, 129.2, 128.5, 126.0, 124.7, 117.5, 115.3, 108.1, 107.6, 83.2, 53.6, 45.7, 34.6, 21.0; HRMS (ESI) *m*/*z* calc’d for C_26_H_23_NO_3_ [M + H]^+^: 398.1751, found (ESI^+^) 398.1754.

9-(2-Hydroxyphenethyl)-2-phenyl-9,10-dihydrochromeno[8,7-*e*][1,3]oxazin-4(8*H*)-one (**6p**). White solid, 67% yield, m.p. 159–162 °C; ^1^H NMR (400 MHz, CDCl_3_) *δ* 10.01 (s, 1H), 8.08 (d, *J* = 8.8 Hz, 1H), 7.85 (d, *J* = 6.0 Hz, 2H), 7.57 (d, *J* = 5.8 Hz, 3H), 7.23 (t, *J* = 7.4 Hz, 1H), 7.10 (d, *J* = 7.4 Hz, 1H), 6.97 (dd, *J* = 12.0, 8.7 Hz, 2H), 6.87 (t, *J* = 7.3 Hz, 1H), 6.81 (s, 1H), 5.04 (s, 2H), 4.47 (s, 2H), 3.25–3.15 (m, 2H), 3.04–2.93 (m, 2H); ^13^C NMR (101 MHz, DMSO-*d*_6_) *δ* 177.0, 162.210, 159.1, 155.6, 154.5, 132.1, 131.8, 130.8, 129.6, 127.6, 126.6, 126.2, 123.9, 119.3, 117.1, 115.4, 115.4, 109.3, 107.2, 83.3, 51.8, 45.1, 29.3; HRMS (ESI) *m*/*z* calc’d for C_25_H_21_NO_4_ [M + H]^+^: 400.1544, found (ESI^+^) 400.1547.

9-(4-Hydroxyphenethyl)-2-phenyl-9,10-dihydrochromeno[8,7-*e*][1,3]oxazin-4(8*H*)-one (**6q**). White solid, 55% yield, m.p. 76–80 °C; ^1^H NMR (400 MHz, DMSO-*d_6_*) *δ* 9.13 (s, 1H), 8.08–8.01 (m, 2H), 7.80 (d, *J* = 8.7 Hz, 1H), 7.63–7.56 (m, 3H), 7.03 (d, *J* = 8.2 Hz, 2H), 6.97–6.89 (m, 2H), 6.64 (d, *J* = 8.3 Hz, 2H), 5.04 (s, 2H), 4.32 (s, 2H), 3.01–2.84 (m, 2H), 2.76 (t, *J* = 7.3 Hz, 2H); ^13^C NMR (101 MHz, DMSO-*d*_6_) *δ* 177.0, 162.3, 159.2, 156.0, 154.5, 132.1, 131.7, 130.2, 130.0, 129.6, 126.7, 123.9, 117.1, 115.4, 115.4, 109.3, 107.2, 83.2, 53.5, 45.3, 33.8; HRMS (ESI) *m*/*z* calc’d for C_25_H_21_NO_4_ [M + H]^+^: 400.1544, found (ESI^+^) 400.1549.

2-(4-Oxo-2-phenylchromeno[8,7-e][1,3]oxazin-9(4*H*,8*H*,10*H*)-yl)acetic acid (**6r**). White solid, 38% yield, m.p. 242–248 °C; ^1^H NMR (400 MHz, DMSO-*d*_6_) *δ* 10.95 (s, 1H), 8.10–8.03 (m, 2H), 7.89 (d, *J* = 8.8 Hz, 1H), 7.64–7.56 (m, 3H), 7.03 (d, *J* = 8.7 Hz, 1H), 6.95 (s, 1H), 4.74 (s, 2H), 3.35 (s, 2H), 3.34 (s, 2H); ^13^C NMR (101 MHz, DMSO-*d*_6_) *δ* 177.2, 165.2, 162.3, 161.8, 156.7, 132.1, 131.9, 129.7, 126.5, 126.5, 116.5, 114.9, 112.1, 110.0, 106.8, 62.4, 58.0; HRMS (ESI) *m*/*z* calc’d for C_19_H_15_NO_5_ [M + H]^+^: 338.1023, found (ESI^+^) 338.1019.

### 4.2. Biological Assays

Each test was repeated three times at 25 ± 1 °C. Active effect expressed in percentage scale of 0–100 (0: no activity; 100: total inhibited). Specific test methods for the anti-TMV and fungicidal activities were carried out by the literature method [30,31]. Detailed bioassay procedures for the anti-TMV and fungicidal activities were described in the literature and can be seen in the Supplementary Materials.

## 5. Conclusions

In this paper, a series of oxazinyl flavonoids were synthesized on the basis of natural product flavone. The anti-TMV activity in vivo and antifungal activity in vitro were evaluated. The structure–activity relationship of antiviral and antifungal activity was discussed by the integration of oxazinyl with the A rings of the mother nucleus structure to protect -OH on the 7-positions and the introduction of different substituents on N-positions of an oxazine ring. In vivo anti-TMV activity showed that most of the compounds showed moderate-to-excellent antiviral activities against TMV at 500 μg/mL. In particular, compound **6p** (inactivation activity, 63%; curative activity, 61%; protection activity, 62%) even exhibited slightly higher anti-TMV activity than ningnanmycin (inactivation activity, 60%; curative activity, 55%; protection activity, 57%) at 500 μg/mL, which was significantly higher than that of ribavirin (inactivation activity, 39%; curative activity, 38%; protection activity, 39%). Antiviral mechanism research by molecular docking exhibited that compounds **6n** and **6p** could interact with TMV CP and inhibit virus assembly. In vitro antifungal activities showed that these oxazinyl flavonoids had broad-spectrum fungicidal activities. In addition, compounds **6h** exhibited antifungal activity of up to 91% against *Physalospora piricola* and might become a candidate drug for new fungicides.

## Figures and Tables

**Table 1:** In vivo antiviral activity of compounds **5** and **6a**–**6r** against TMV.

Compd.	Anti-TMV Activities (%) ^a^		
	Inactivation Effect	Curative Effect	Protection Effect
**5**	33 ± 1	30 ± 1	30 ± 1
**6a**	38 ± 3	31 ± 3	36 ± 3
**6b**	41 ± 2	40 ± 1	43 ± 1
**6c**	34 ± 1	31 ± 2	33 ± 3
**6d**	48 ± 2	43 ± 3	46 ± 2
**6e**	41 ± 3	31 ± 1	34 ± 2
**6f**	36 ± 2	34 ± 3	35 ± 3
**6g**	30 ± 2	25 ± 3	28 ± 3
**6h**	24 ± 1	23 ± 3	25 ± 3
**6i**	21 ± 3	20 ± 3	21 ± 3
**6j**	43 ± 3	38 ± 3	44 ± 2
**6k**	40 ± 2	42 ± 1	40 ± 1
**6l**	23 ± 3	21 ± 3	23 ± 3
**6m**	18 ± 2	18 ± 3	19 ± 3
**6n**	62 ± 1	59 ± 1	60 ± 1
**6o**	53 ± 1	50 ± 1	51 ± 1
**6p**	63 ± 1	61 ± 1	62 ± 1
**6q**	45 ± 2	44 ± 1	42 ± 3
**6r**	0	0	0
Apigenin	28 ± 1	25 ± 1	26 ± 1
Ribavirin	39 ± 2	38 ± 3	39 ± 2
Ningnanmycin	60 ± 1	55 ± 2	57 ± 1

^a^ Average of three replicates. All results are expressed as mean ± SD.

**Table 2:** In vitro fungicidal activities of compounds **5** and **6a**–**6r** against 6 kinds of fungi.

Compd.	Fungicidal Activity (%) ^a^ at 50 μg/mL					
	*F.c.* ^b^	*C.h.* ^b^	*P.p.* ^b^	*R.c.* ^b^	*P.g.* ^b^	*S.s.* ^b^
**5**	26 ± 3	21 ± 2	49 ± 1	25 ± 2	42 ± 1	71 ± 2
**6a**	47 ± 1	23 ± 2	80 ± 1	55 ± 1	20 ± 3	43 ± 1
**6b**	36 ± 1	26 ± 1	10 ± 1	50 ± 3	71 ± 1	44 ± 3
**6c**	35 ± 2	28 ± 2	74 ± 1	55 ± 1	33 ± 1	25 ± 3
**6d**	22 ± 1	23 ± 2	12 ± 2	11 ± 3	28 ± 2	65 ± 2
**6e**	19 ± 1	23 ± 2	21 ± 2	33 ± 1	20 ± 3	7 ± 2
**6f**	21 ± 2	21 ± 3	58 ± 2	49 ± 1	7 ± 2	36 ± 2
**6g**	24 ± 2	21 ± 3	61 ± 1	57 ± 2	7 ± 2	25 ± 3
**6h**	26 ± 1	21 ± 3	91 ± 1	26 ± 2	33 ± 1	43 ± 1
**6i**	23 ± 1	21 ± 3	44 ± 3	31 ± 3	20 ± 3	43 ± 1
**6j**	52 ± 2	52 ± 2	27 ± 1	50 ± 3	43 ± 1	78 ± 1
**6k**	19 ± 1	21 ± 3	69 ± 2	52 ± 1	7 ± 2	39 ± 1
**6l**	19 ± 1	28 ± 2	54 ± 1	33 ± 1	20 ± 3	21 ± 1
**6m**	31 ± 2	28 ± 2	76 ± 2	39 ± 1	20 ± 3	36 ± 2
**6n**	29 ± 2	34 ± 2	10 ± 1	55 ± 1	43 ± 1	52 ± 2
**6o**	18 ± 2	18 ± 1	22 ± 3	39 ± 3	71 ± 1	74 ± 1
**6p**	18 ± 2	21 ± 2	7 ± 1	50 ± 3	71 ± 1	78 ± 1
**6q**	23 ± 2	18 ± 1	31 ± 2	31 ± 2	71 ± 1	54 ± 1
**6r**	24 ± 2	33 ± 1	50 ± 3	39 ± 1	7 ± 2	32 ± 2
Apigenin	3 ± 2	16 ± 1	46 ± 1	50 ± 3	43 ± 1	48 ± 1
Chlorothalonil ^c^	100	74 ± 2	100	91 ± 1	89 ± 1	100
Carbendazim ^c^	92 ± 1	52 ± 1	100	90 ± 2	100	100

^a^ Average of three replicates. All results are expressed as mean ± SD. ^b^ F.c., *Fusarium oxysporium* f. sp. Cucumeris; C.h., *Cercospora arachidicola* Hori; P.p., *Physalospora piricola*; R.c., *Rhizoctonia cerealis*; P.g., *Pyricularia grisea*; S.s., *Sclerotinia sclerotiorum*. ^c^ The commercial agricultural fungicides were used for comparison of antifungal activity.

## Data Availability

All data used to support the findings of this study are included within the article and Supplementary Materials.

## Supplementary Materials

The following supporting information can be downloaded at https://www.mdpi.com/article/10.3390/molecules27206875/s1, Section S1: Detailed bio-assay procedures; Section S2: Calculation procedures for molecular docking research; Section S3: Copies of NMR spectra (Figures S1–S44). References [30,35,40,41,42] were cited in supplementary materials.

## Sample Availability

Samples of the compounds **2**−**5** and **6a**−**6r** are available from the authors.
