# Supplementary material for: Synthesis and Biological Activity of Novel Oxazinyl Flavonoids as Antiviral and Anti-Phytopathogenic Fungus Agents
Source: Molecules. 2022 Oct 13;27(20):6875. doi: 10.3390/molecules27206875 (PMC9611556; doi:10.3390/molecules27206875)
Supplement: Supplementary file 1 [file molecules-27-06875-s001.zip › molecules-1937654-supplementary.pdf]

Supplementary Material

Synthesis and Biological Activity of Novel Oxazinyl Flavonoids as Antiviral and Anti-Phytopathogenic Fungus Agents

Yucong Ma †, Lu Wang †, Aidang Lu * and Wei Xue *

School of Chemical Engineering and Technology, Hebei University of Technology, Tianjin 300401, China

* Correspondence: luaidang@hebut.edu.cn (A.L.); weixue@hebut.edu.cn (W.X.); Tel.: +86-22-60202812 (A.L.); +86-22-60202419 (W.X.)

† These two authors contributed equally to this work.

Contents

Section S1: Detailed bio-assay procedures 3

1.1 Phytotoxic Activity 3

1.2 Antiviral Biological Assay 3

1.3 Detailed bioassay procedures for the fungicidal activities 3

Section S2: Calculation procedures for molecular docking research 4

Section S3: Copies of NMR spectra (Figures S1–S44) 5

Figures S1–S2. ^{1}H NMR and ^{13}C NMR spectra of **2** 5

Figures S3–S4. ^{1}H NMR and ^{13}C NMR spectra of **3** 6

Figures S5–S6. ^{1}H NMR and ^{13}C NMR spectra of **4** 7

Figures S7–S8. ^{1}H NMR and ^{13}C NMR spectra of **5** 8

Figures S9–S10. ^{1}H NMR and ^{13}C NMR spectra of **6a** 9

Figures S11–S12. ^{1}H NMR and ^{13}C NMR spectra of **6b** 10

Figures S13–S14. ^{1}H NMR and ^{13}C NMR spectra of **6c** 11

Figures S15–S16. ^{1}H NMR and ^{13}C NMR spectra of **6d** 12

Figures S17–S18. ^{1}H NMR and ^{13}C NMR spectra of **6e** 13

Figures S19–S20. ^{1}H NMR and ^{13}C NMR spectra of **6f** 14

Figures S21–S22. ^{1}H NMR and ^{13}C NMR spectra of **6g** 15

Figures S23–S24. ^{1}H NMR and ^{13}C NMR spectra of **6h** 16

Figures S25–S26. ^{1}H NMR and ^{13}C NMR spectra of **6i** 17

Figures S27–S28. ^{1}H NMR and ^{13}C NMR spectra of **6j** 18

Figures S29–S30. ^{1}H NMR and ^{13}C NMR spectra of **6k** 19

Figures S31–S32. ^{1}H NMR and ^{13}C NMR spectra of **6l** 20

Figures S33–S34. ^{1}H NMR and ^{13}C NMR spectra of **6m** 21

Figures S35–S36. ^{1}H NMR and ^{13}C NMR spectra of **6n** 22

Figures S37–S38. ^{1}H NMR and ^{13}C NMR spectra of **6o** 23

Figures S39–S40. ^{1}H NMR and ^{13}C NMR spectra of **6p** 24

Figures S41–S42. ^{1}H NMR and ^{13}C NMR spectra of **6q** 25

Figures S43–S44. ^{1}H NMR and ^{13}C NMR spectra of **6r** 26

Section 1. The detailed bio-assay procedures

1.1. Phytotoxic Activity.

The growing 5–6 leaf stage tobaccos (*Nicotiana tabacum var Xanthi nc*) were selected. The compound solution (500 μg/mL) was smeared on the leaves and calculated the number of lesions after 0, 3, 7 and 10 days respectively. [1] There are three replicates for each compound.

1.2. Antiviral Biological Assay

1.2.1. Purification of Tobacco Mosaic Virus.

Using Gooding's method [2], the upper leaves of *Nicotiana tabacum* L. inoculated with TMV were selected and ground in phosphate buffer and then filtered through double-layer pledget. The filtrate was centrifuged at 10000 g, treated with PEG twice, and centrifuged again. The whole experiment was processed at 4 °C. Absorbance value was estimated at 260 nm by ultraviolet spectrophotometer.

$$Virus\ concn = (A_{260} \times dilution\ ratio)\ E/_{1cm}^{0.1\%,260nm}$$

1.2.2. Protective Effect of Compounds against TMV in Vivo.

The compound solution was smeared on the left side and the solvent serving as control on the right side of growing *N. tabacum* L. leaves of the same ages. The leaves were then inoculated with the virus after 12 h. A brush was dipped in TMV of 6×10^{-3} mg/mL to inoculate the leaves, which were previously scattered with silicon carbide. The leaves were then washed with water and rubbed softly along the nervature once or twice. The local lesion numbers appearing 3–4 days after inoculation were counted. [1,3] There are three replicates for each compound.

1.2.3. Inactivation Effect of Compounds against TMV in Vivo.

The virus was inhibited by mixing with the compound solution at the same volume for 30 min. The mixture was then inoculated on the left side of the leaves of *N. tabacum* L., whereas the right side of the leaves was inoculated with the mixture of solvent and the virus for control. The local lesion numbers were recorded 3–4 days after inoculation. [1,3] There are three replicates for each compound.

1.2.4. Curative Effect of Compounds against TMV in Vivo.

Growing leaves of *N. tabacum* L. of the same ages were selected. TMV (concentration of 6.0×10^{-3} mg/mL) was dipped and inoculated on the whole leaves. Then the leaves were washed with water and dried. The compound solution was smeared on the left side, and the solvent was smeared on the right side for control. The local lesion numbers were then counted and recorded 3–4 days after inoculation. [1,3] There are three replicates for each compound. The in vitro and in vivo inhibition rates of the compound were then calculated according to the following formula ("av" means average, and controls were not treated with compound).

Inhibition rate (%) = [(av local lesion no. of control—av local lesion no. of drug-treated)/av local lesion no. of control] × 100%

1.3. Detailed bioassay procedures for the fungicidal activities

1.3.1. In Vitro Antifungal Bioassay.

The fungicidal activities of compounds were evaluated in mycelial growth tests conducted in artificial media against 14 plant pathogens at a rate of 50 μg/mL. Each test compound was dissolved in a suitable amount of acetone and diluted with water containing 0.1% TW-80 to a concentration of 500 μg/mL. To each petri dish was added 1 mL of the test solution and 9 mL of culture medium to make a 50 μg/mL concentration of the test compound, while in another petri dish was added 1 mL

distilled water containing 0.1% TW-80 and 9 mL of culture medium as a blank control. A 4 mm diameter of hyphal growth was cut using a hole puncher on a growing fungal culture and the hyphae were moved to the petri dish containing the test compound. Each assay was performed three times. The dishes were stored in controlled environment cabinets (24 ± 1 °C) for 4 days, after which the diameter of mycelial growth was measured and the percentage inhibition was calculated using the following equation: *Percentage inhibition (%)* = (*averaged diameter of mycelia in blank controls—averaged diameter of mycelia in medicated tablets*)/(*averaged diameter of mycelia in blank controls*—4 mm) × 100.[4]

Section 2. Calculation procedures for molecular docking research

The calculation procedures for molecular docking research consist of four steps. [5]

Receptor Preparation. The 3D crystal structure of TMV-CP (PDB code:1EI7) was downloaded from the protein data bank (PDB) and this was used as the receptor for molecular docking. Water molecules were removed from the target protein and hydrogen atoms were added using AutoDock Tools prior to molecular docking.

Ligand preparation. Target compounds are drawn using ChemOffice 2015 as ligands followed by management of its conformer and the minimisation process.

Molecular Docking Using AutoDock Vina. The input files for AutoDock Vina were prepared using AutoDock Tools. The protein was placed in a grid box (grid parameters: center x = 5, center y = -20, center z = 0.8, size x = 60, size y = 60, size z = 56), using AutoDock Vina at 1.00 Å to define the binding site. The docking procedure was performed using the instructed command prompts.

Analysing and Output Visualisation using PyMOL. The docking poses were ranked according to their docking scores. The scoring function in Auto Dock was used to predict the binding affinity of one ligand to the receptor molecule. The conformation with the lowest binding affinity was selected for further analysis after the docking process. The docking results included the locations of hydrogen bonds and closely interacting residues were performed by PyMOL software.

References

1. Wang, Z. W.; Wei, P.; Wang, L. Z.; Wang, Q. M. Design, synthesis, and anti-tobacco mosaic virus (TMV) activity of phenanthroindolizidines and their analogues. *J. Agric. Food Chem.* **2012**, *60*, 10212–10219.
2. Gooding, G. V., Jr.; Hebert, T. T. A simple technique for purification of tobacco mosaic virus in large quantities. *Phytopathology* **1967**, 57, 1285–1290.
3. Li, S. Z.; Wang, D. M.; Jiao, S. M. In Pesticide Experiment Methods-Fungicide Sector; Li, S. Z., Ed.; Agriculture Press of China: Beijing, China, **1991**, 93–94.
4. Zhao, H. P.; Liu, Y. X.; Cui, Z. P.; Beattie, D.; Gu, Y. C.; Wang, Q. M. Design, synthesis, and biological activities of arylmethylamine substituted chlorotriazine and methylthiotriazine compounds. *J. Agric. Food Chem.* **2011**, *59*, 11711–11717.
5. Seyedi, S. S.; Shukri, M.; Hassandarvish, P.; Oo, A.; Muthu, S. E.; Abubakar, S.; Zandi, K. Computational approach towards exploring potential anti-chikungunya activity of selected flavonoids. *Sci. Rep.* **2016**, *6*, 24027.

Section 3. Figures S1–S44. ^{1}**H, and** ^{13}**C NMR spectra of 2–6**

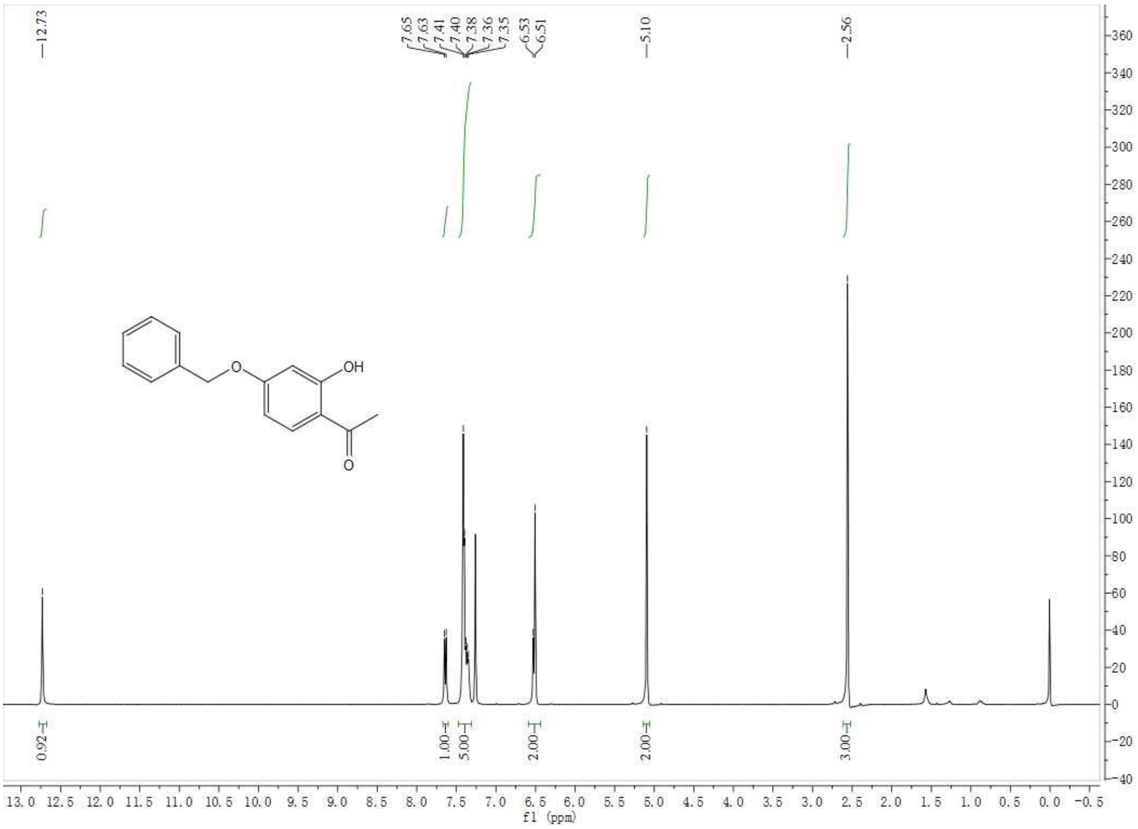

Figure S1. ^{1}H NMR spectrum of **2**

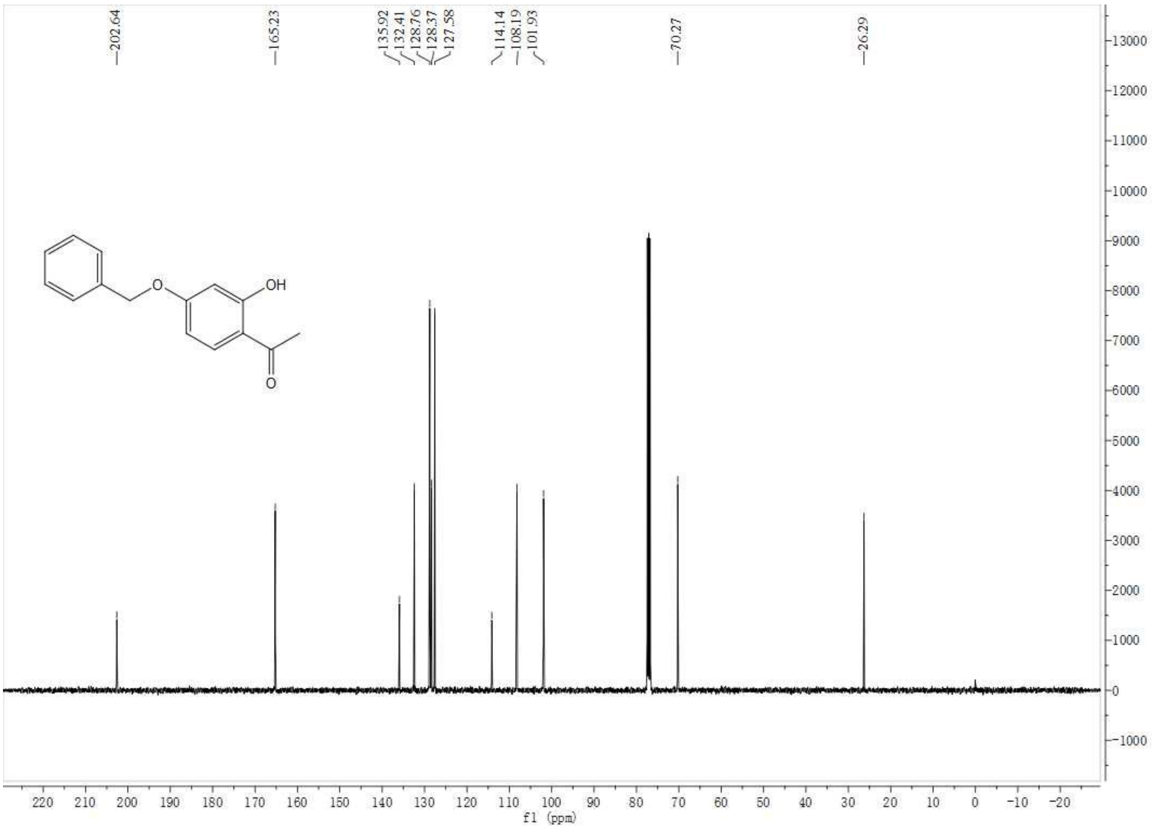

Figure S2. ^{13}C NMR spectrum of **2**

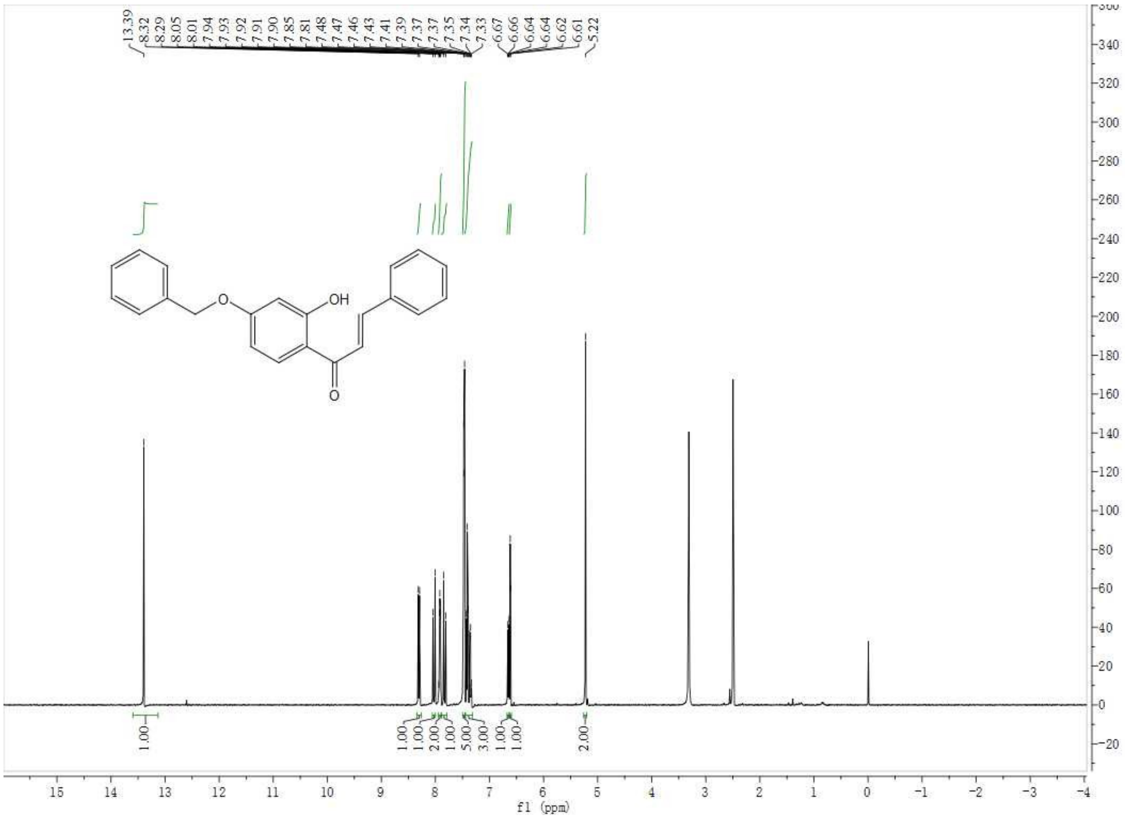

Figure S3. ^{1}H NMR spectrum of **3**

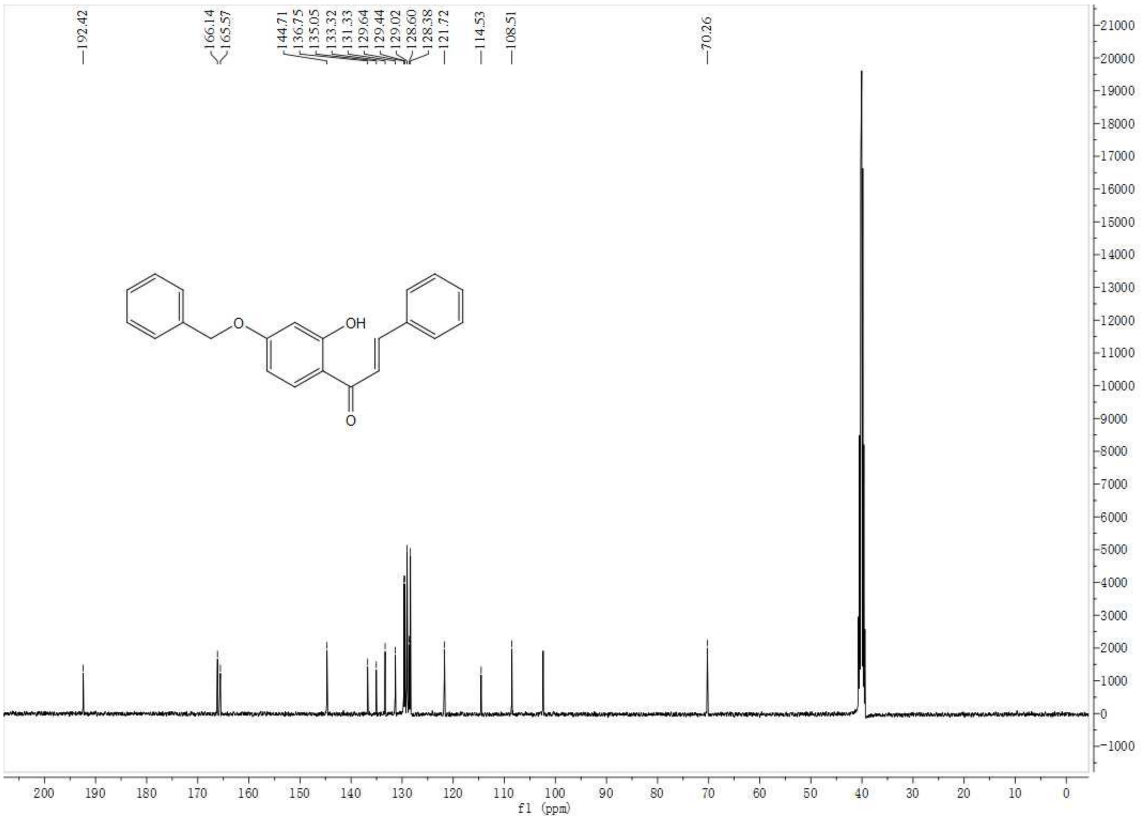

Figure S4. ^{13}C NMR spectrum of **3**

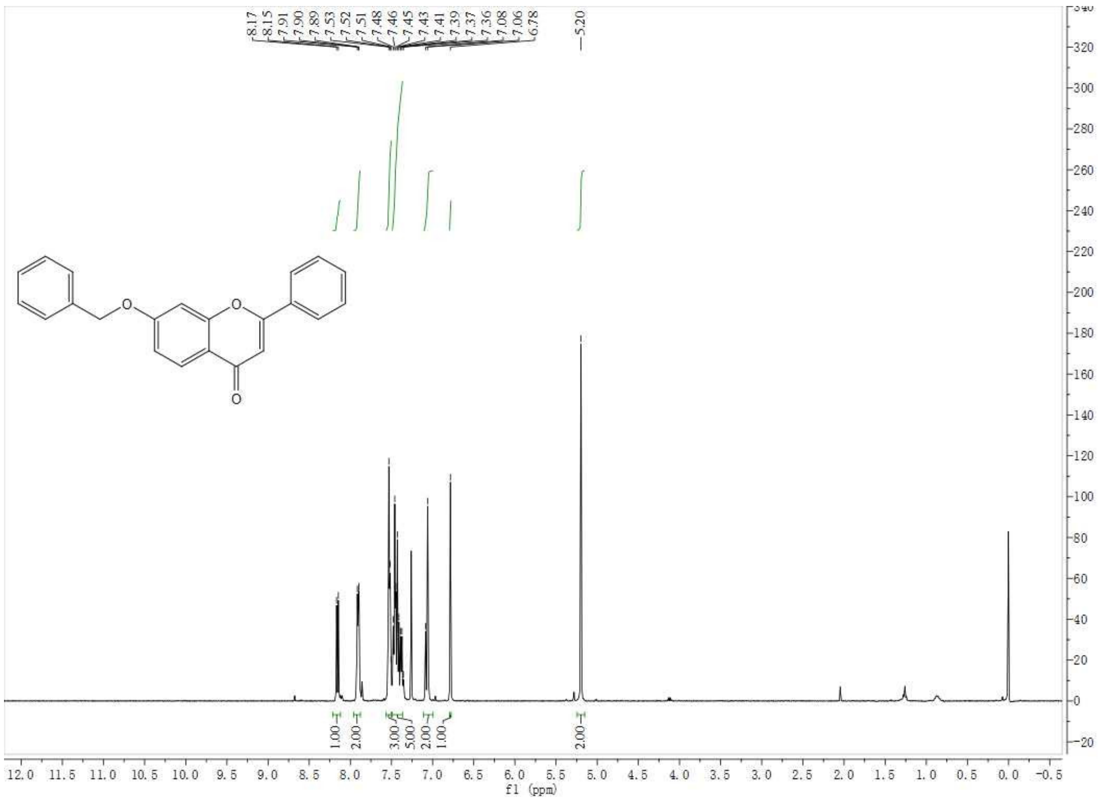

Figure S5. ^{1}H NMR spectrum of **4**

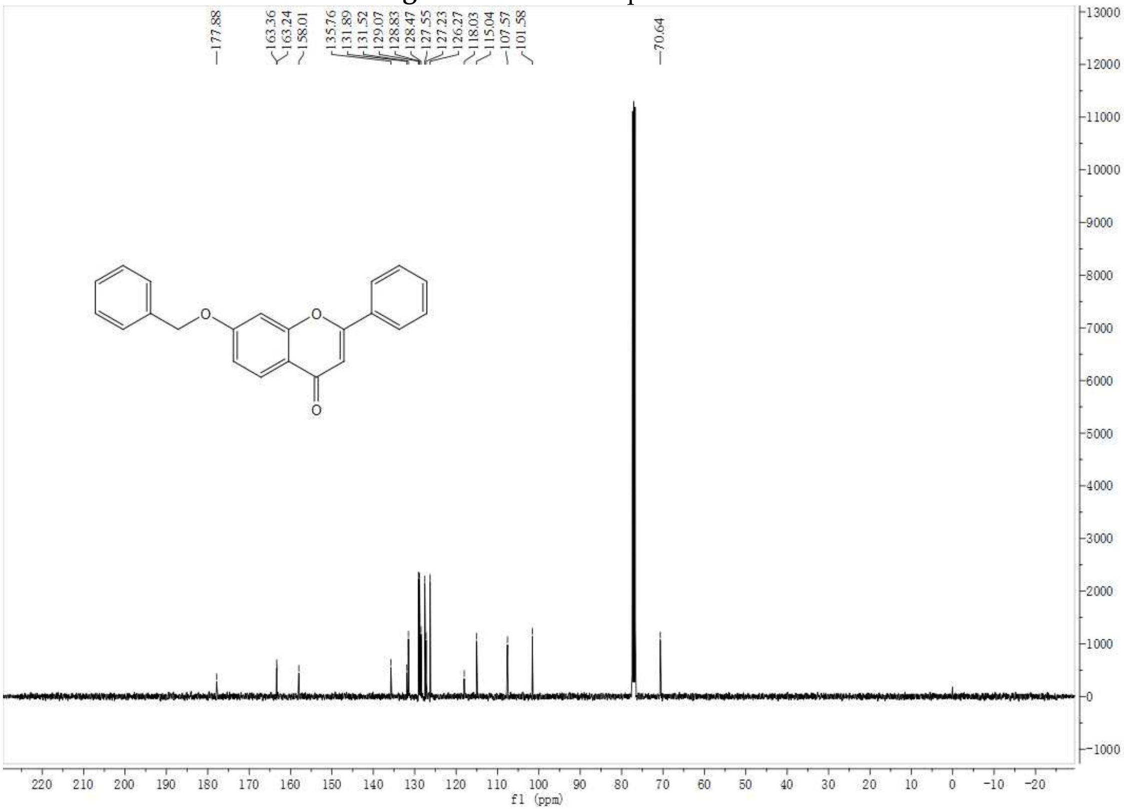

Figure S6. ^{13}C NMR spectrum of **4**

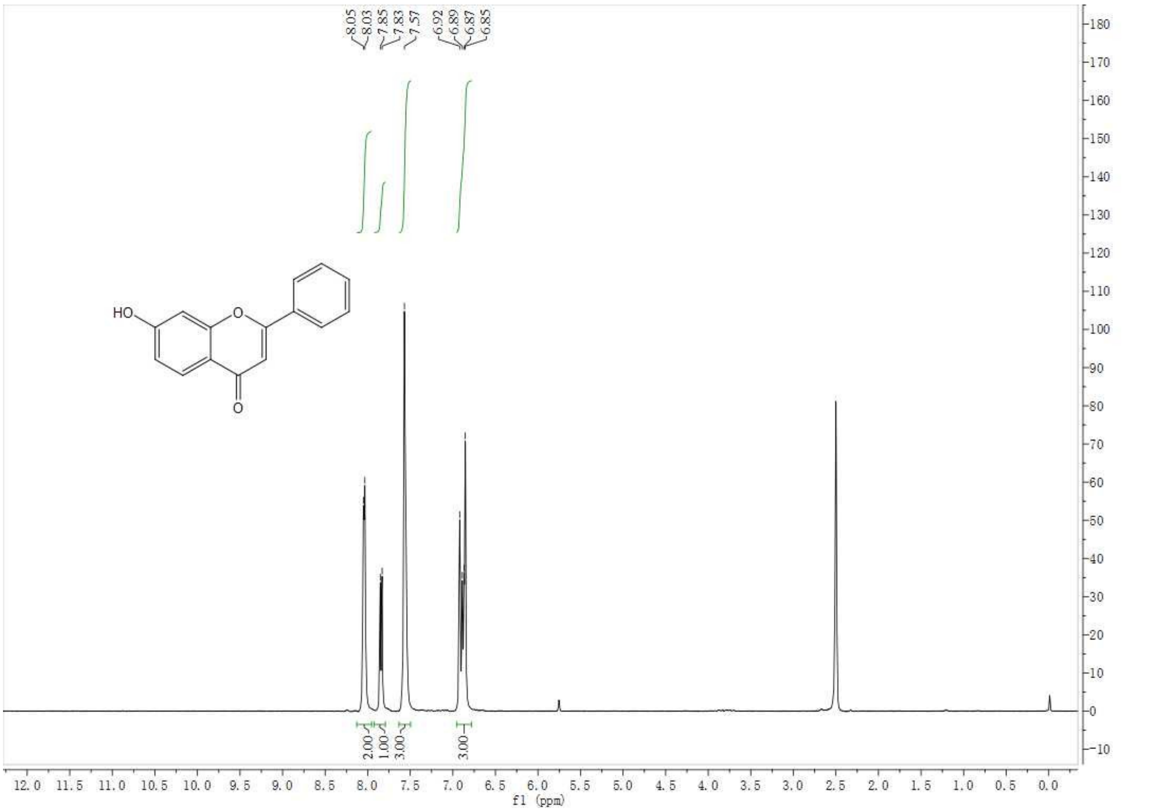

Figure S7. ^{1}H NMR spectrum of **5**

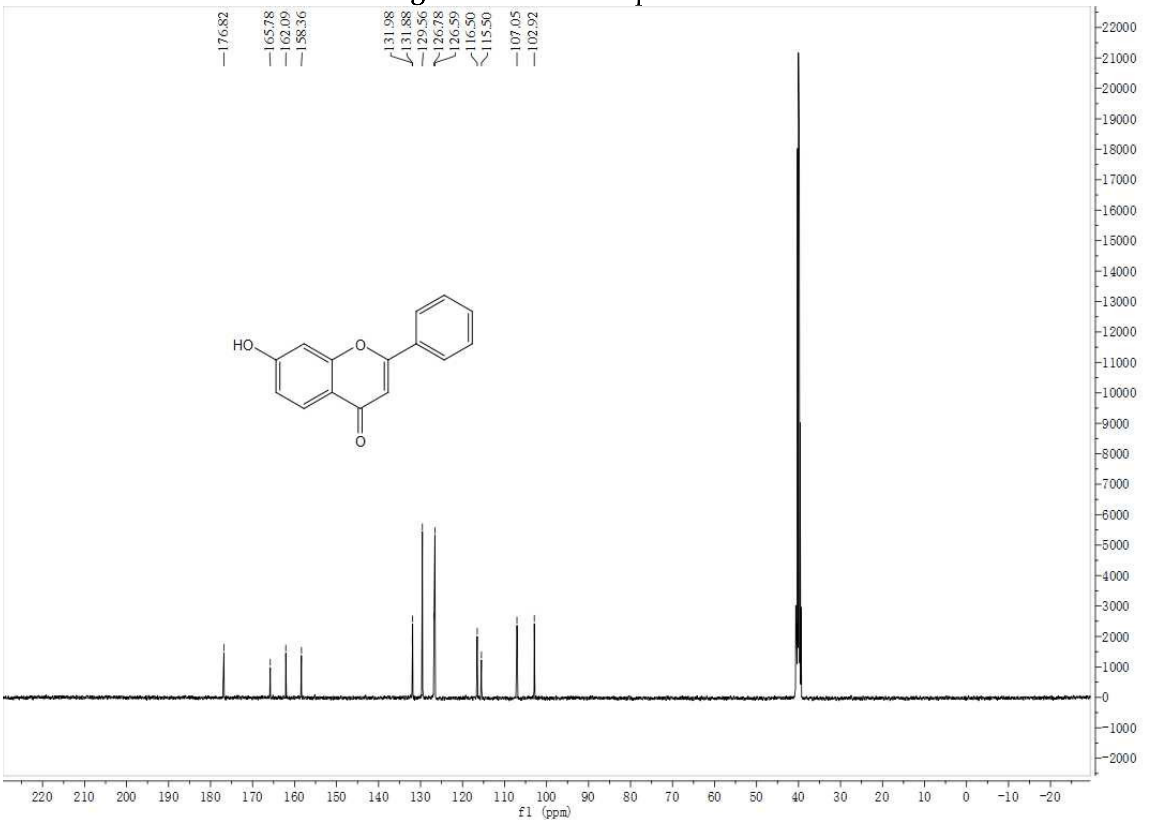

Figure S8. ^{13}C NMR spectrum of **5**

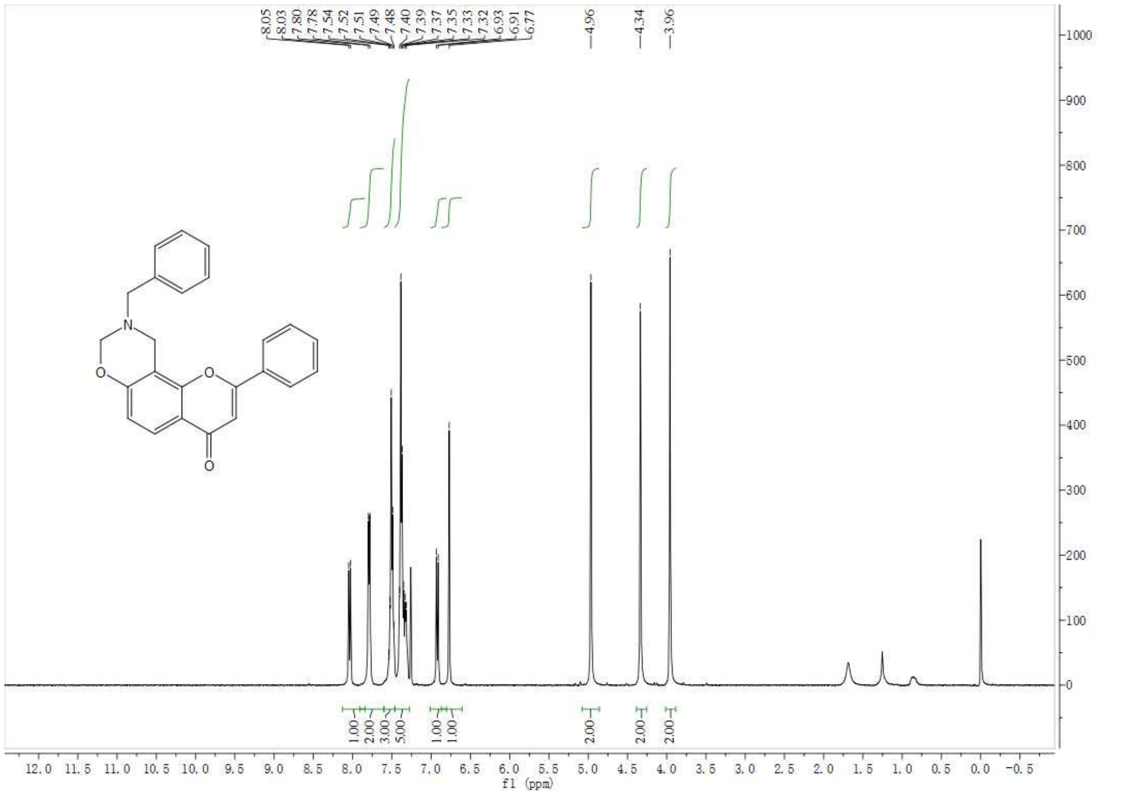

Figure S9. ^{1}H NMR spectrum of **6a**

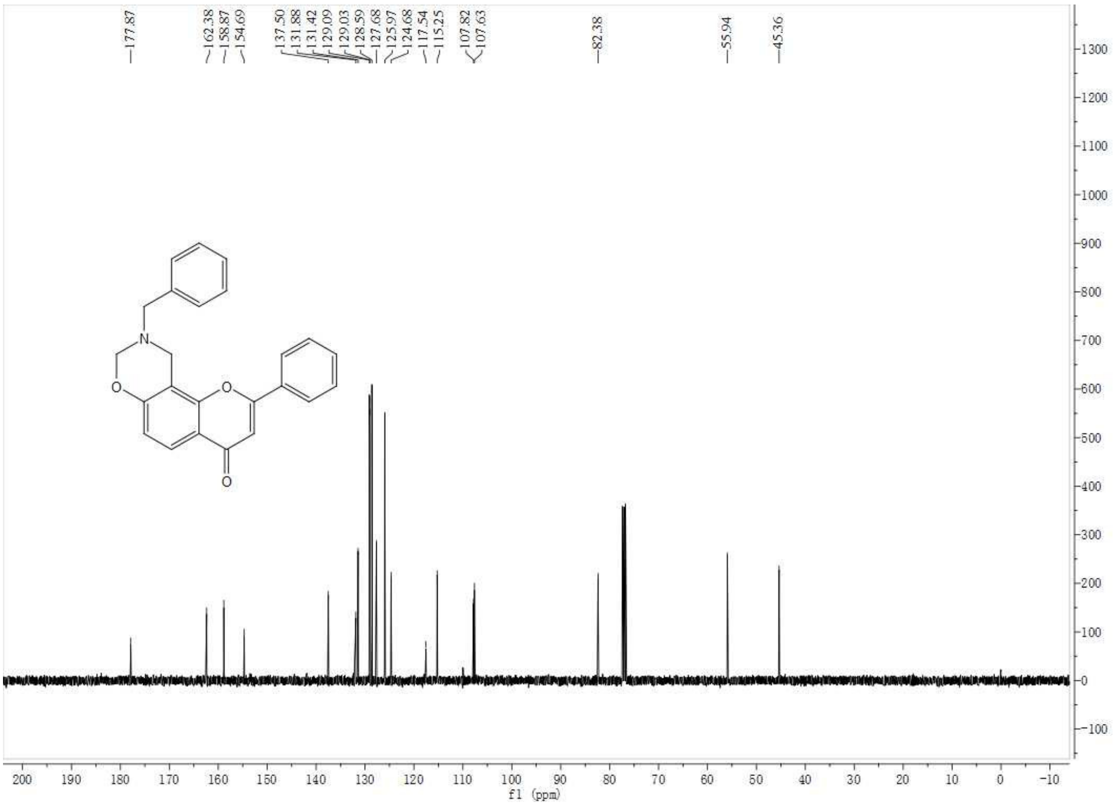

Figure S10. ^{13}C NMR spectrum of **6a**

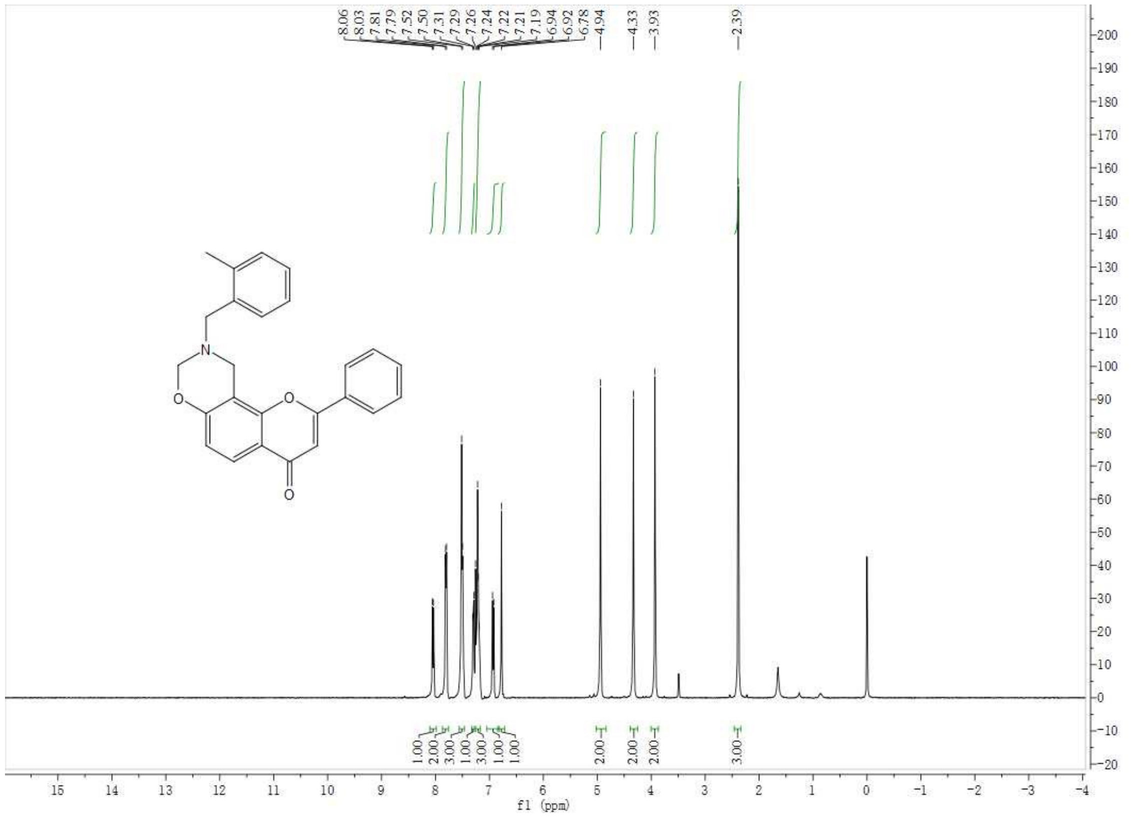

Figure S11. ^{1}H NMR spectrum of **6b**

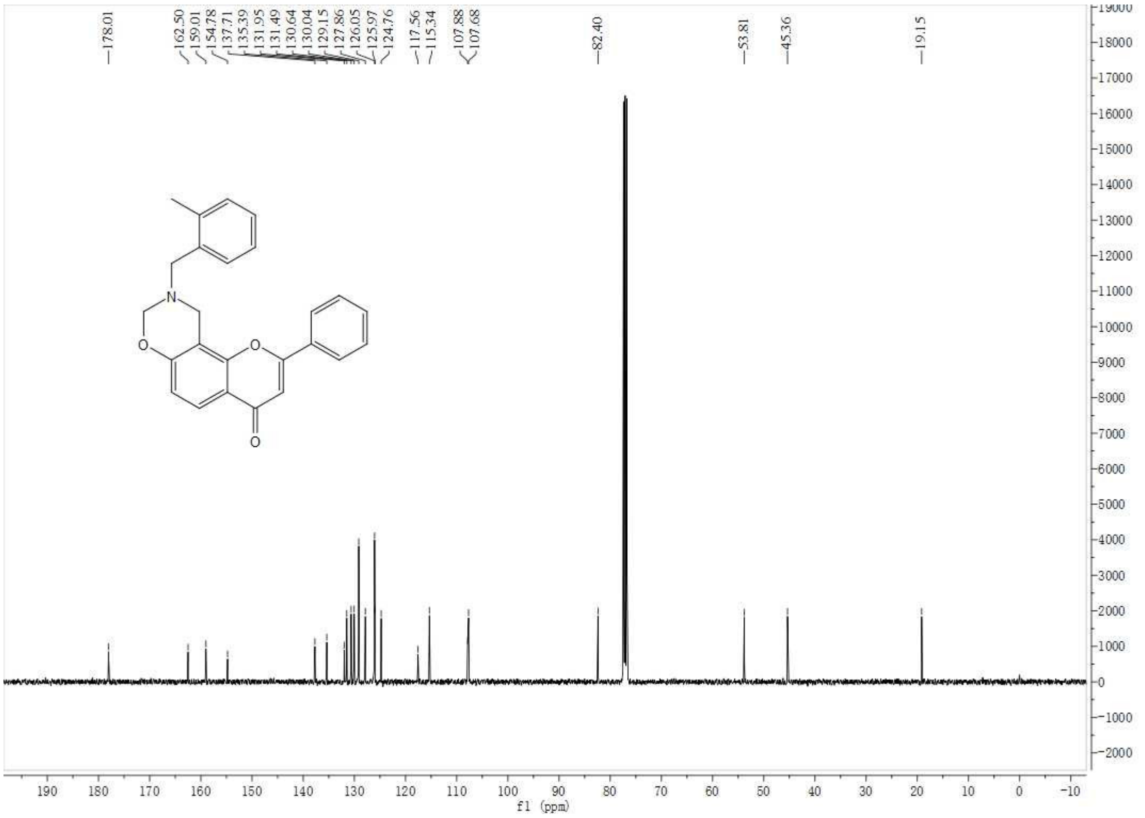

Figure S12. ^{13}C NMR spectrum of **6b**

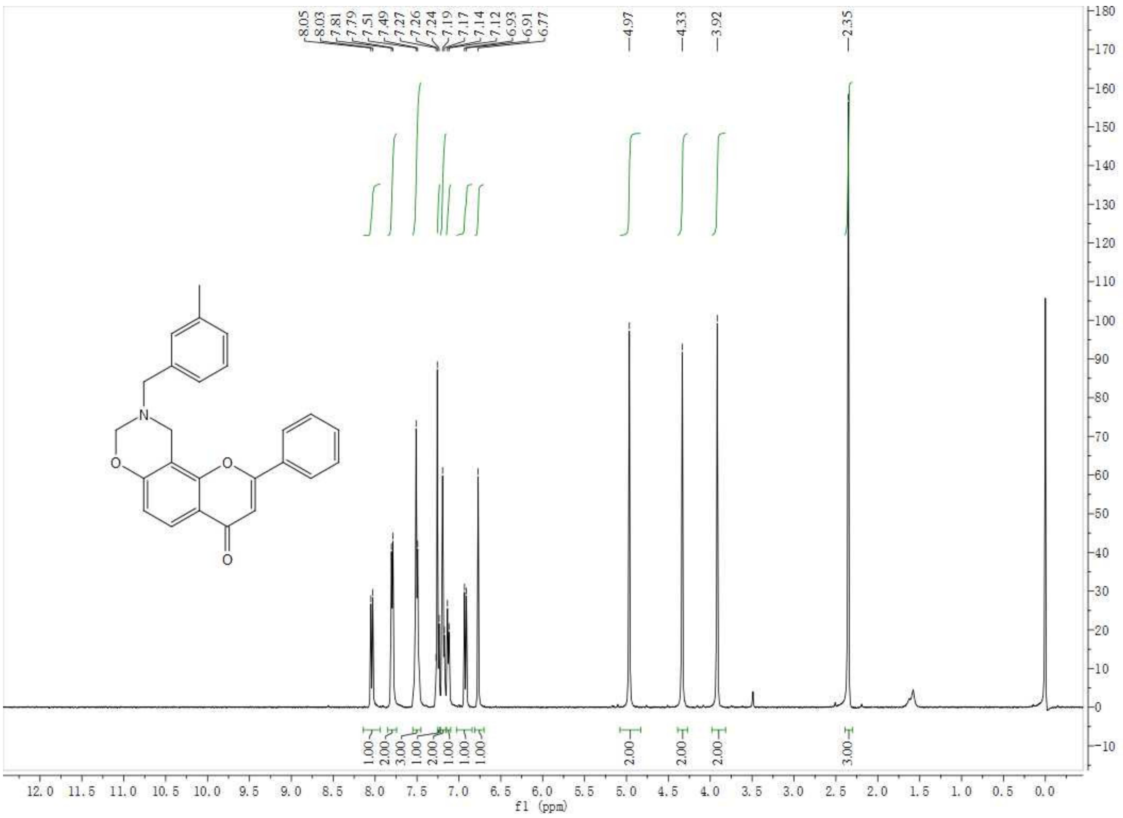

Figure S13. ^{1}H NMR spectrum of **6c**

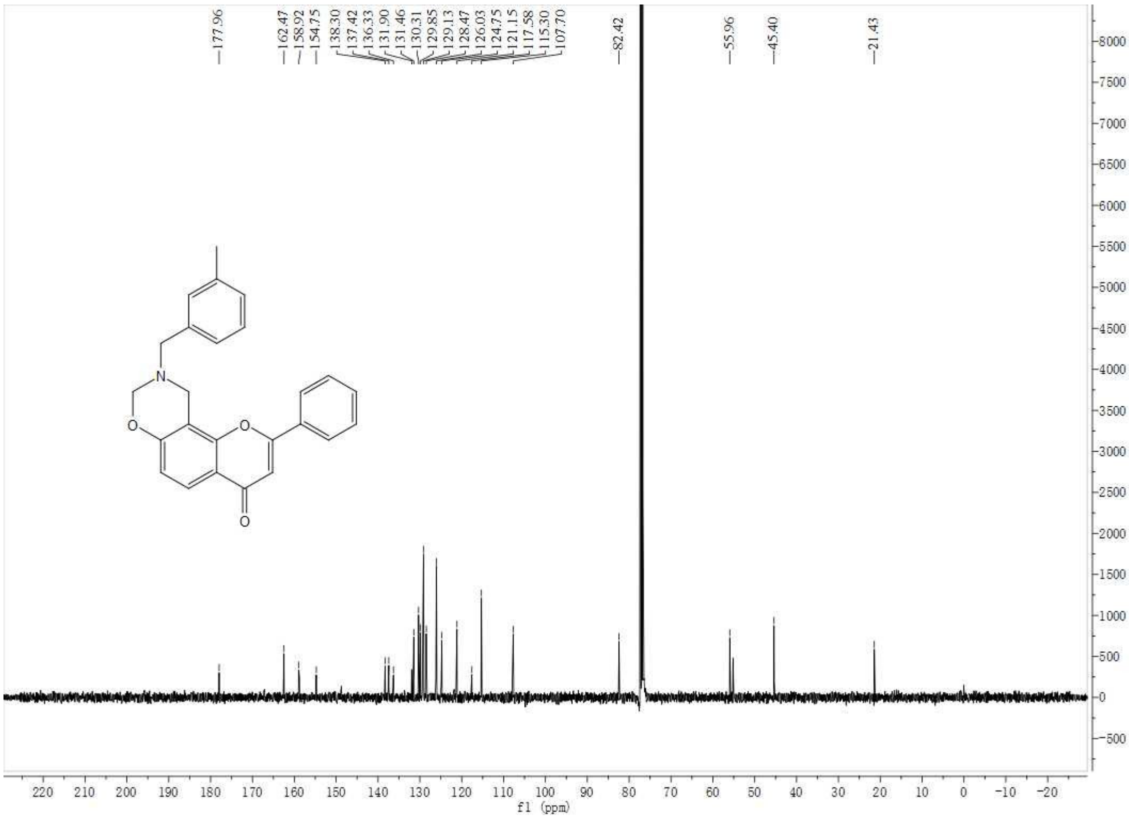

Figure S14. ^{13}C NMR spectrum of **6c**

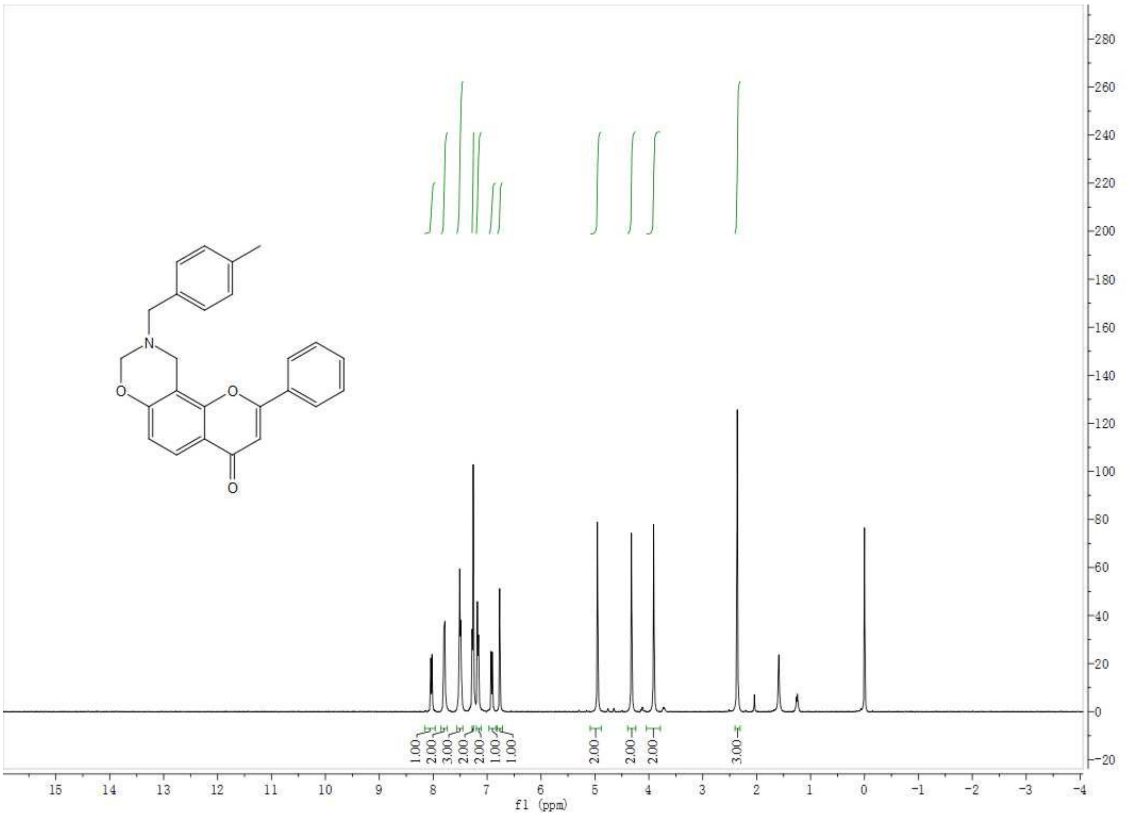

Figure S15. ^{1}H NMR spectrum of **6d**

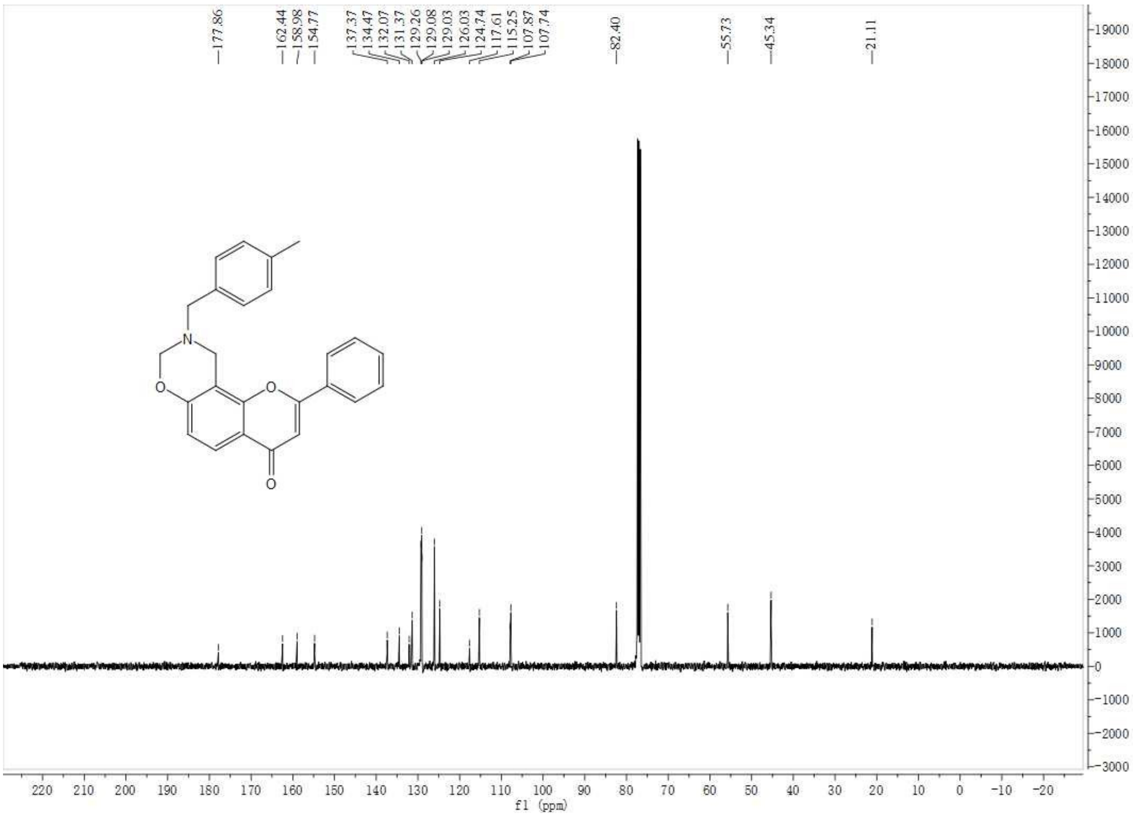

Figure S16. ^{13}C NMR spectrum of **6d**

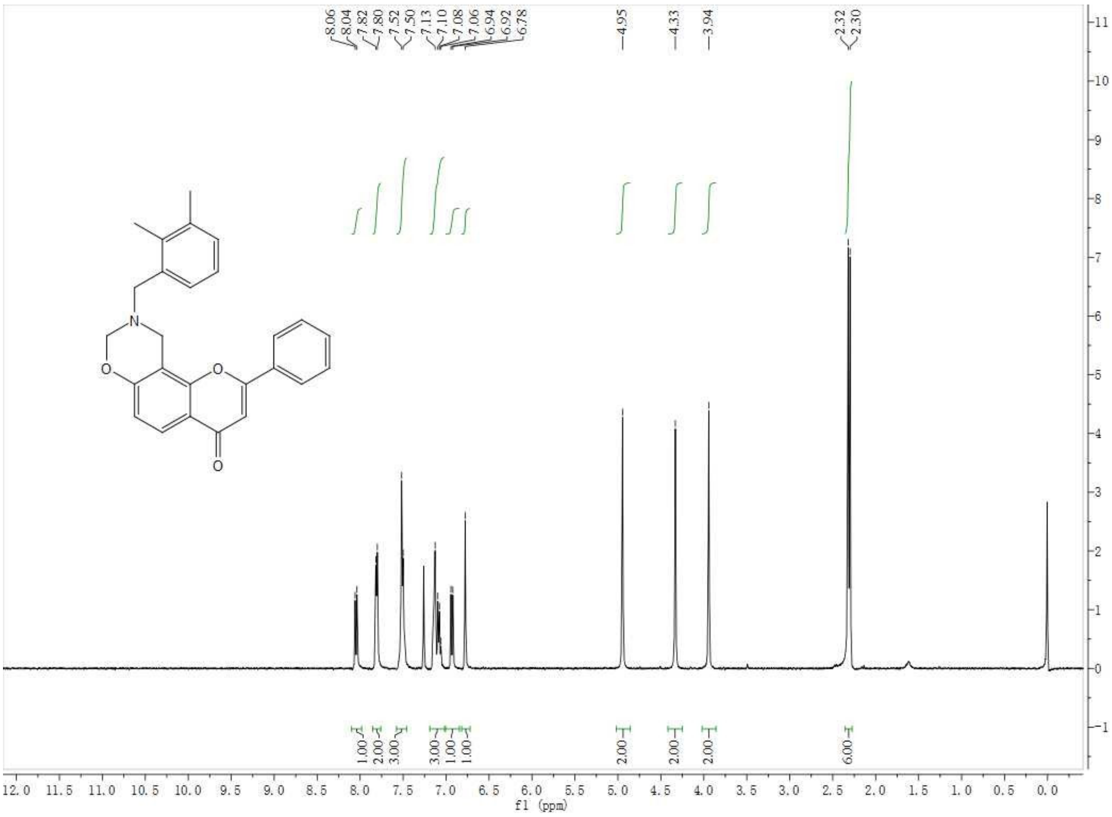

Figure S17. ^{1}H NMR spectrum of **6e**

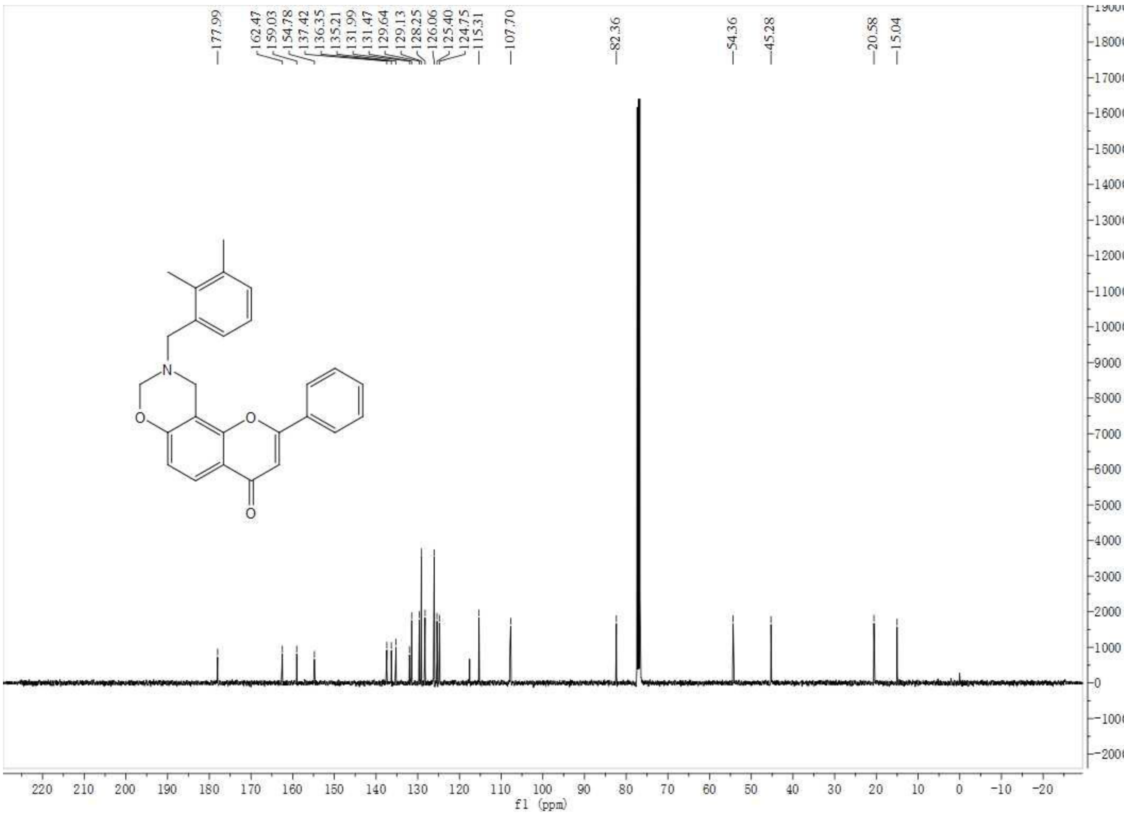

Figure S18. ^{13}C NMR spectrum of **6e**

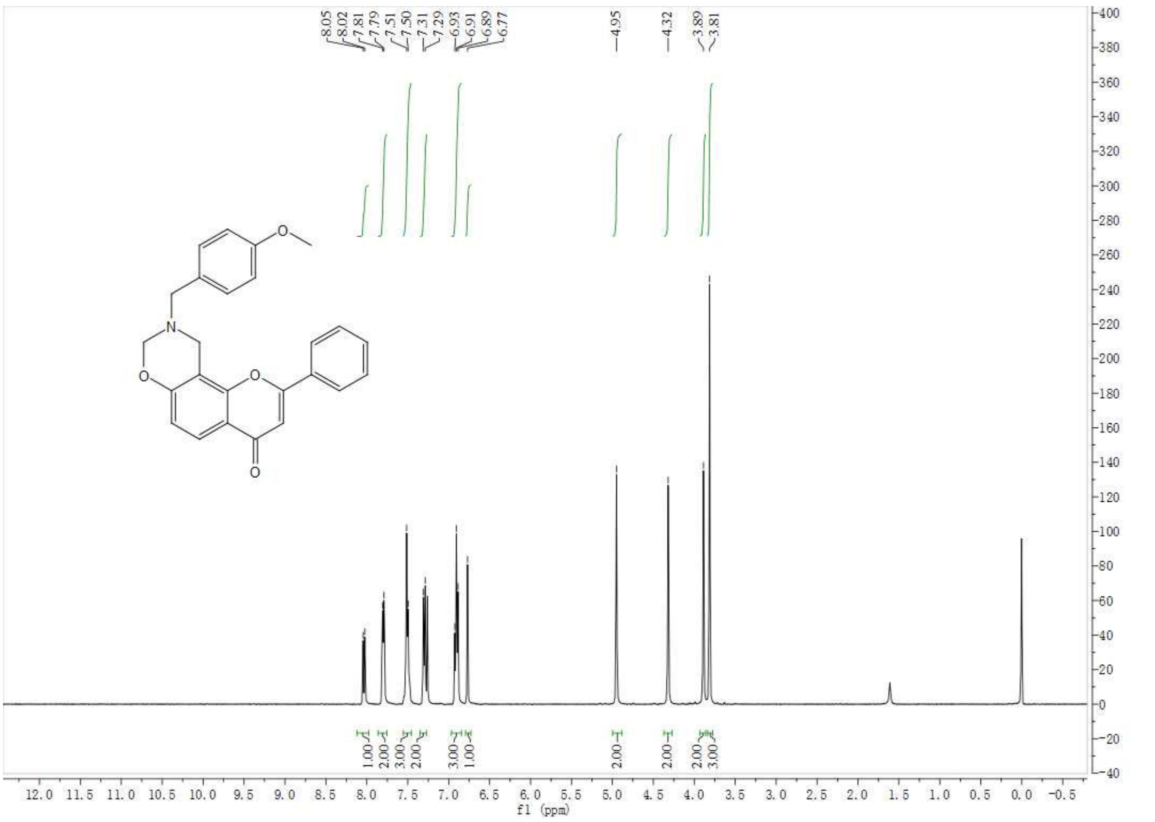

Figure S19. ¹H NMR spectrum of **6f**

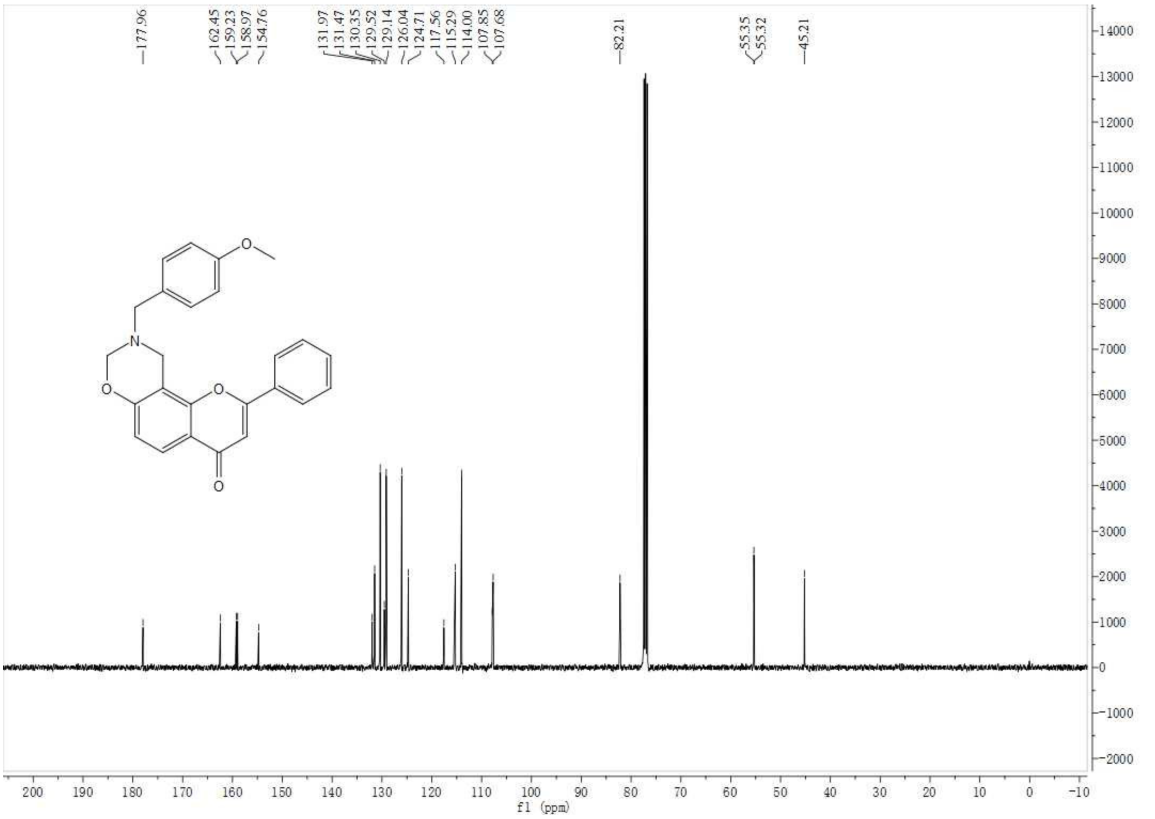

Figure S20. ¹³C NMR spectrum of **6f**

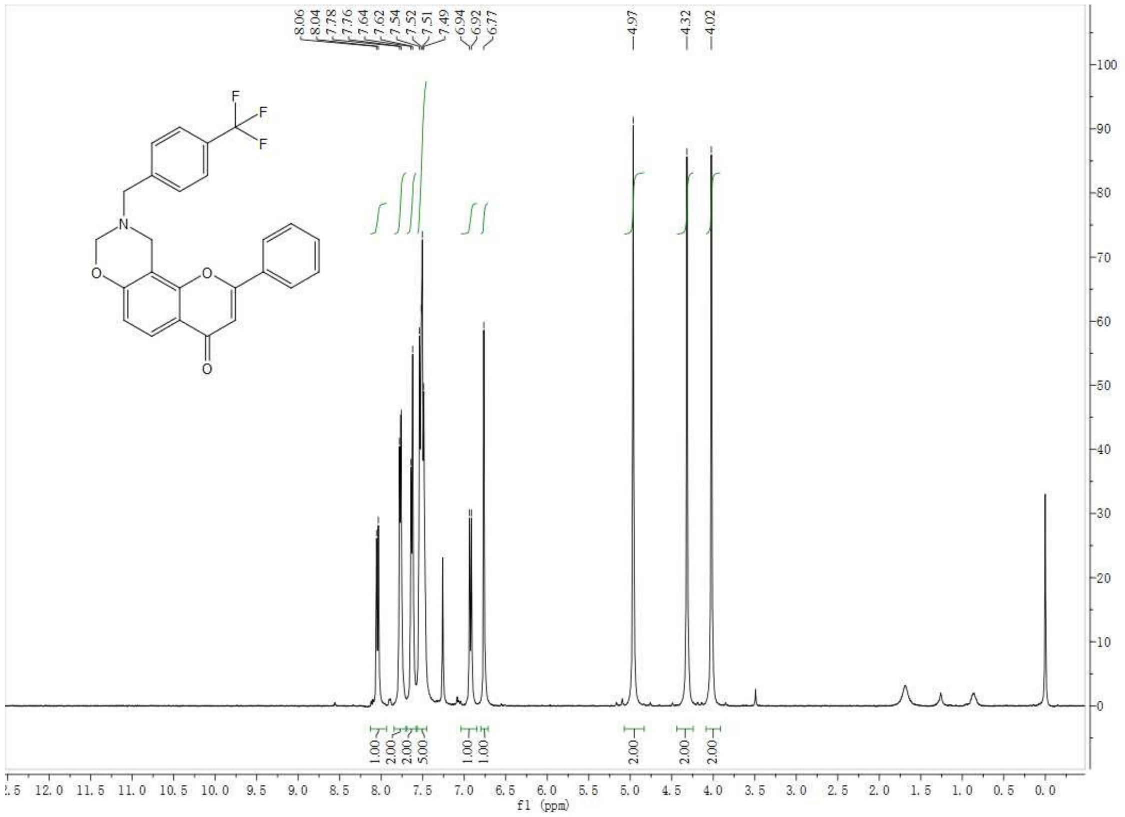

Figure S21. ¹H NMR spectrum of **6g**

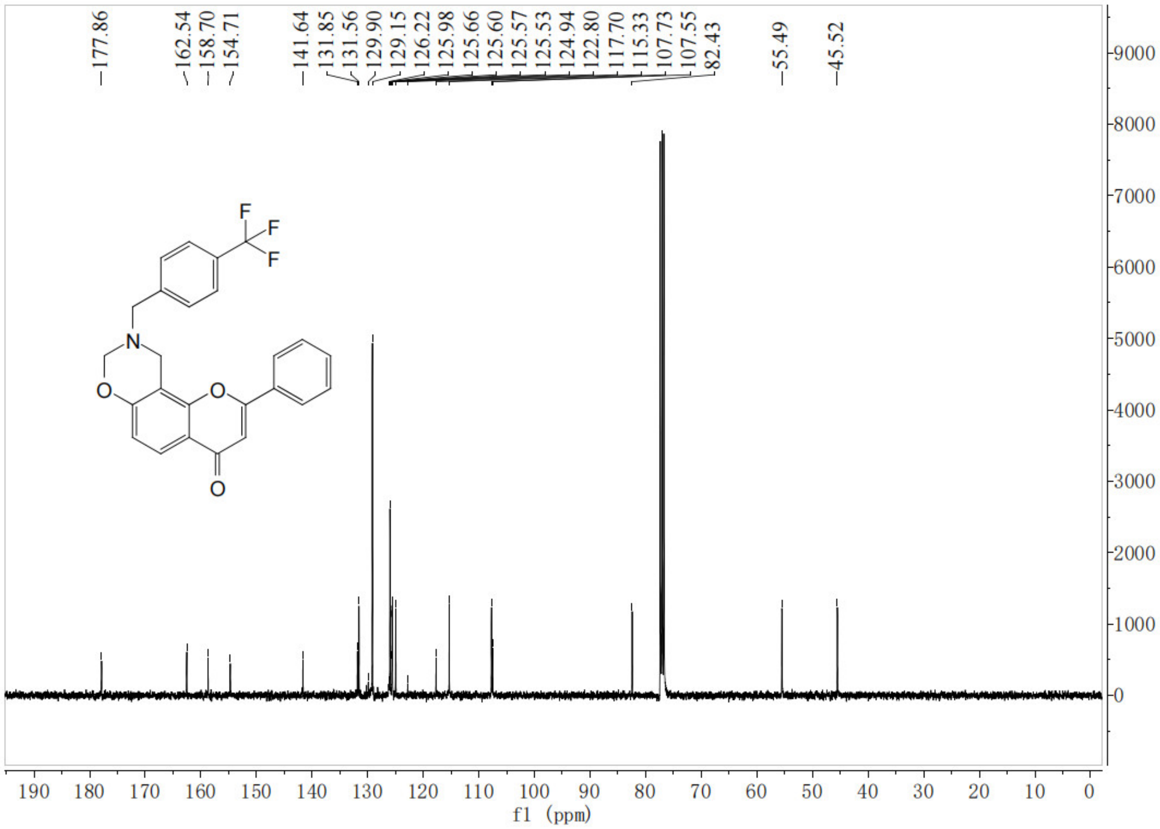

Figure S22. ¹³C NMR spectrum of **6g**

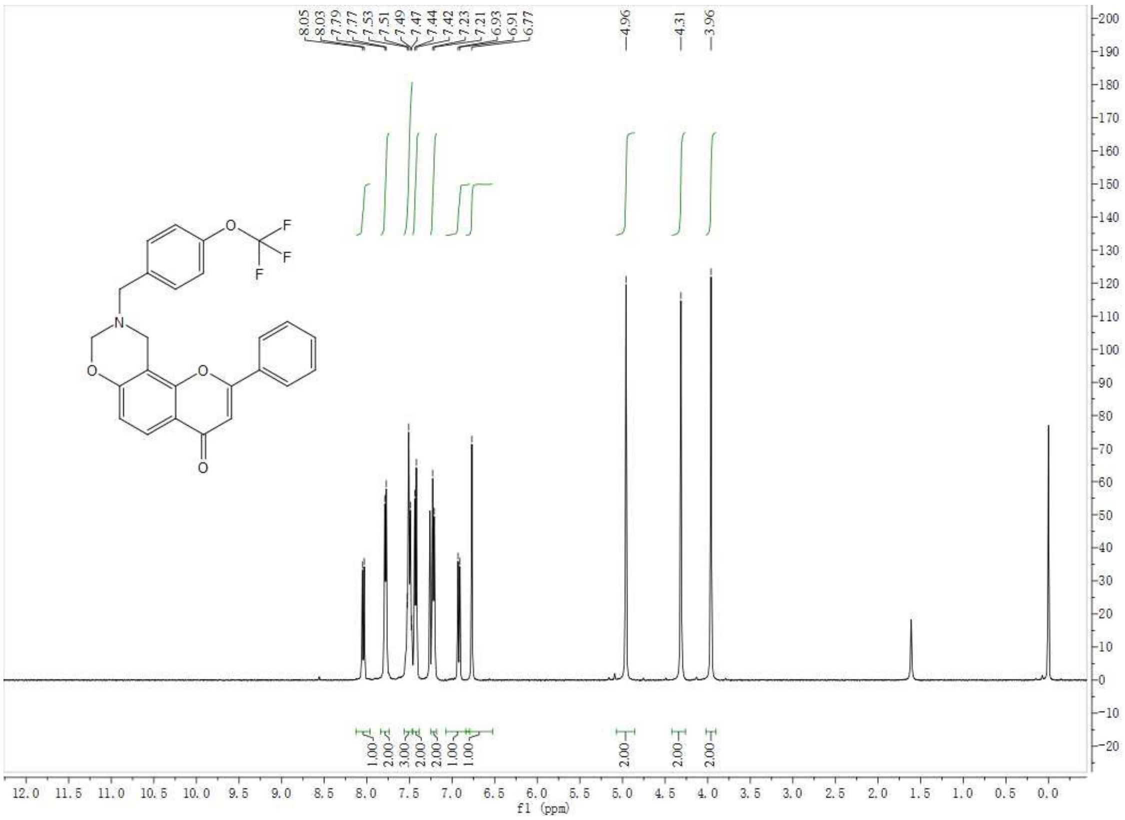

Figure S23. ^{1}H NMR spectrum of **6h**

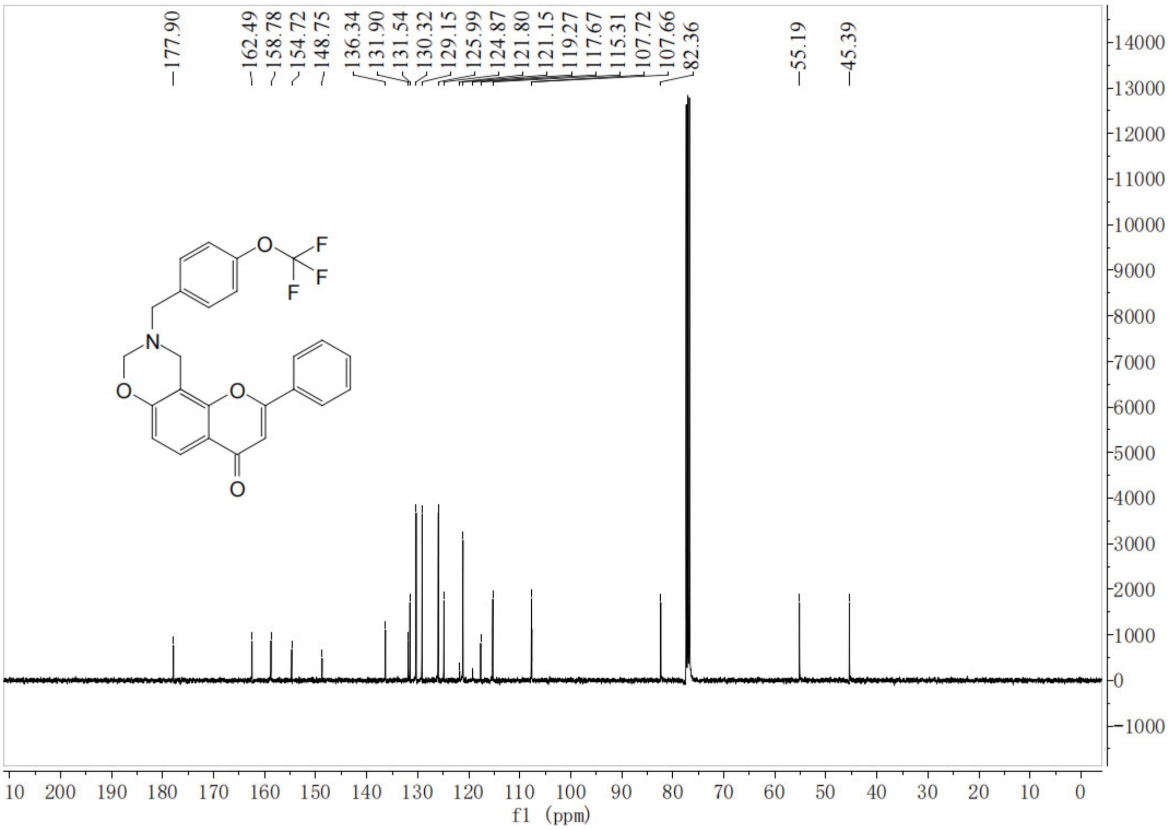

Figure S24. ^{13}C NMR spectrum of **6h**

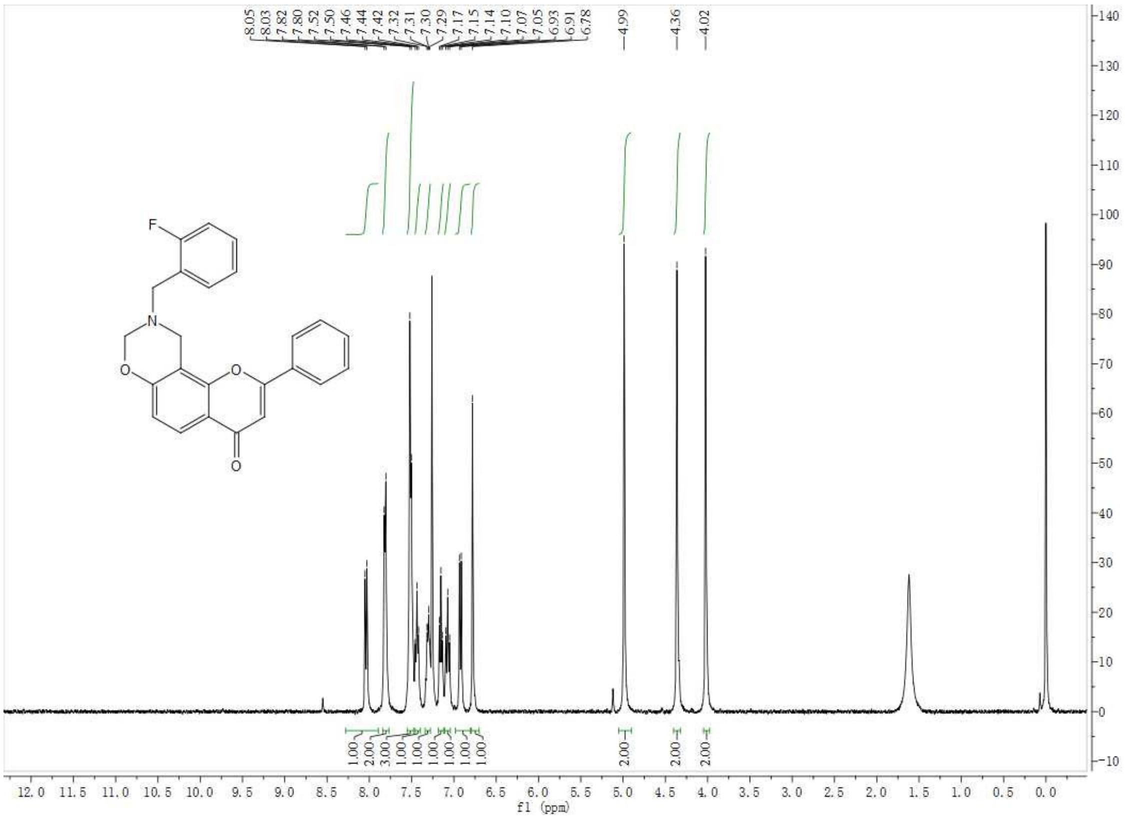

Figure S25. ^{1}H NMR spectrum of **6i**

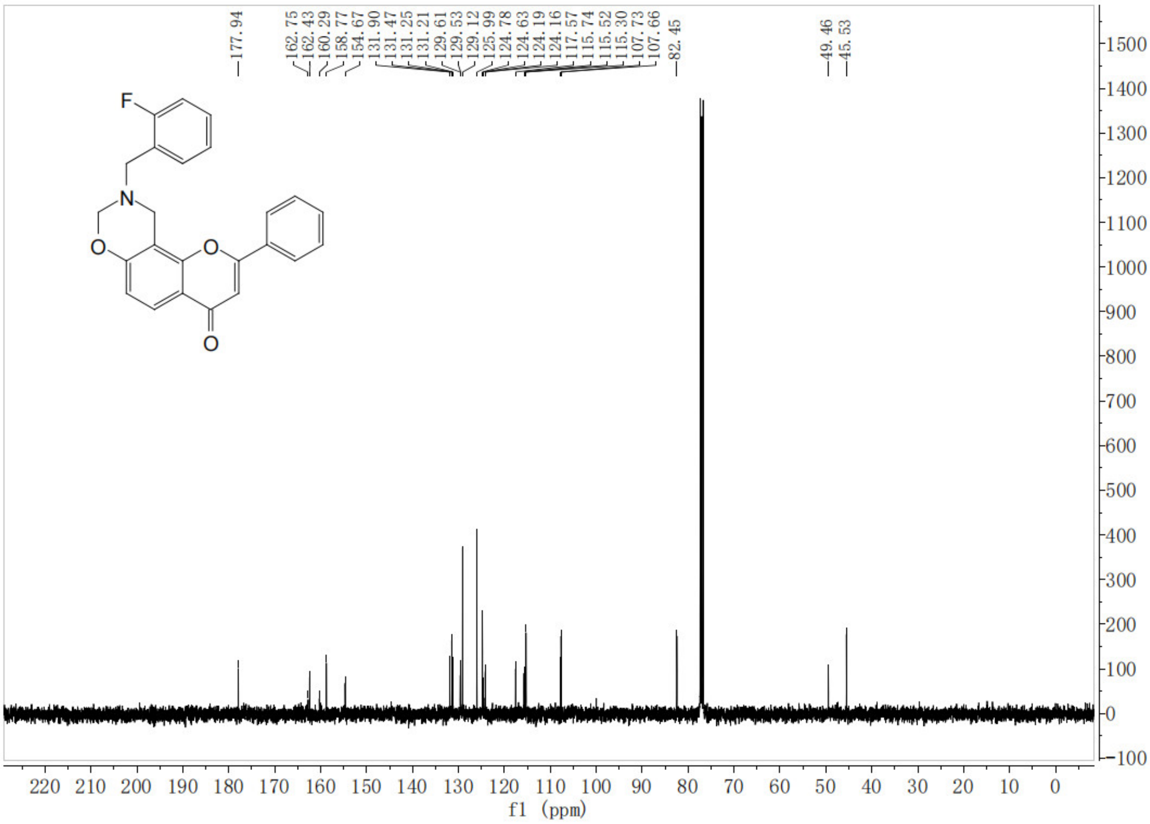

Figure S26. ^{13}C NMR spectrum of **6i**

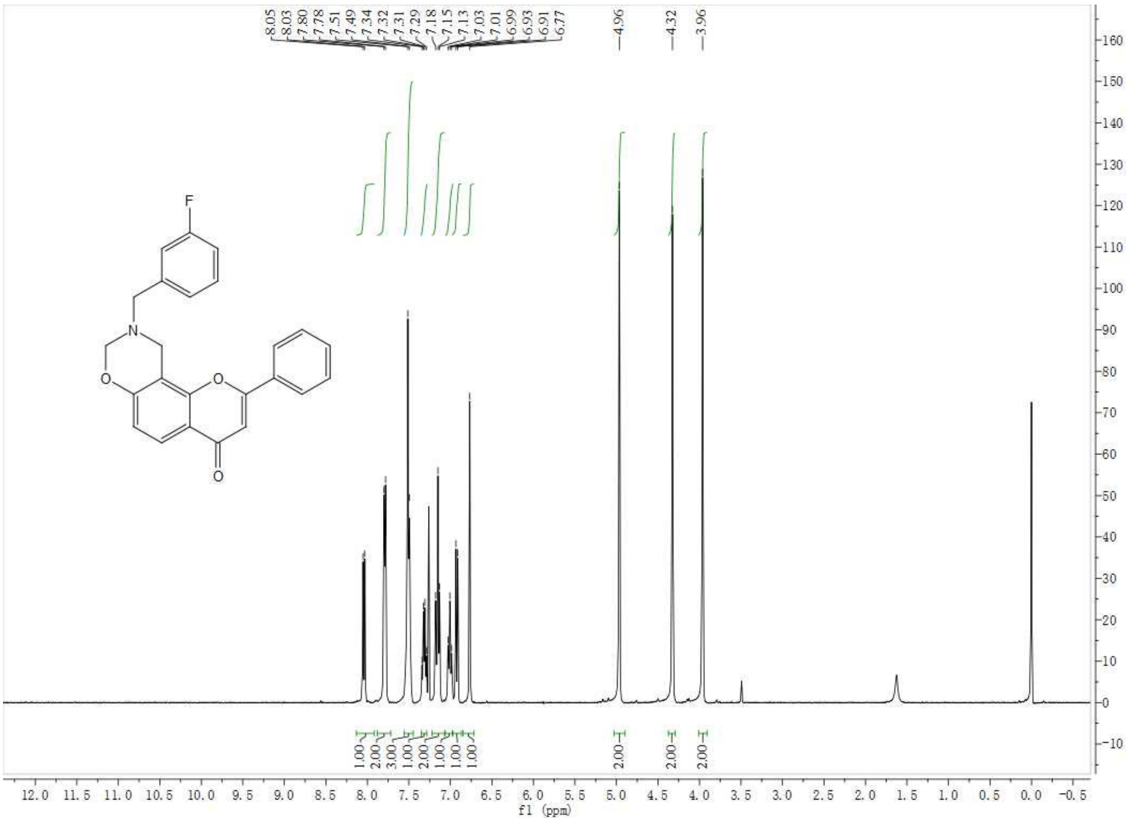

Figure S27. ^{1}H NMR spectrum of **6j**

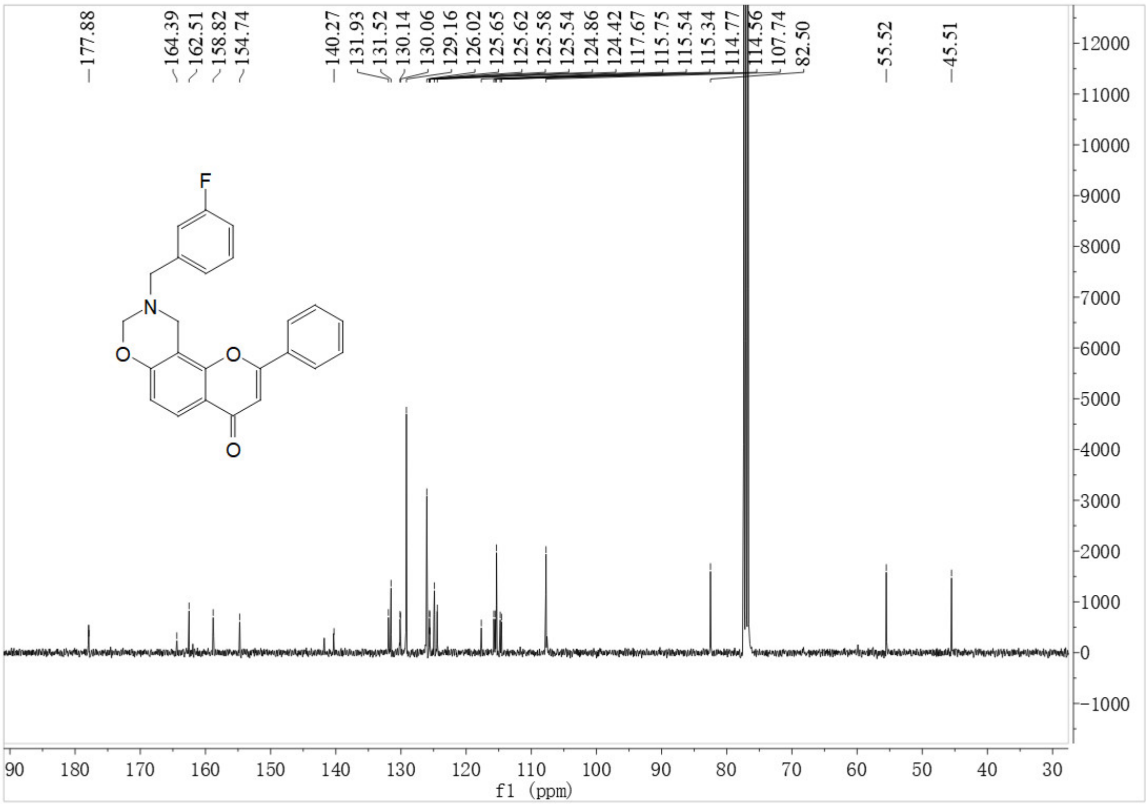

Figure S28. ^{13}C NMR spectrum of **6j**

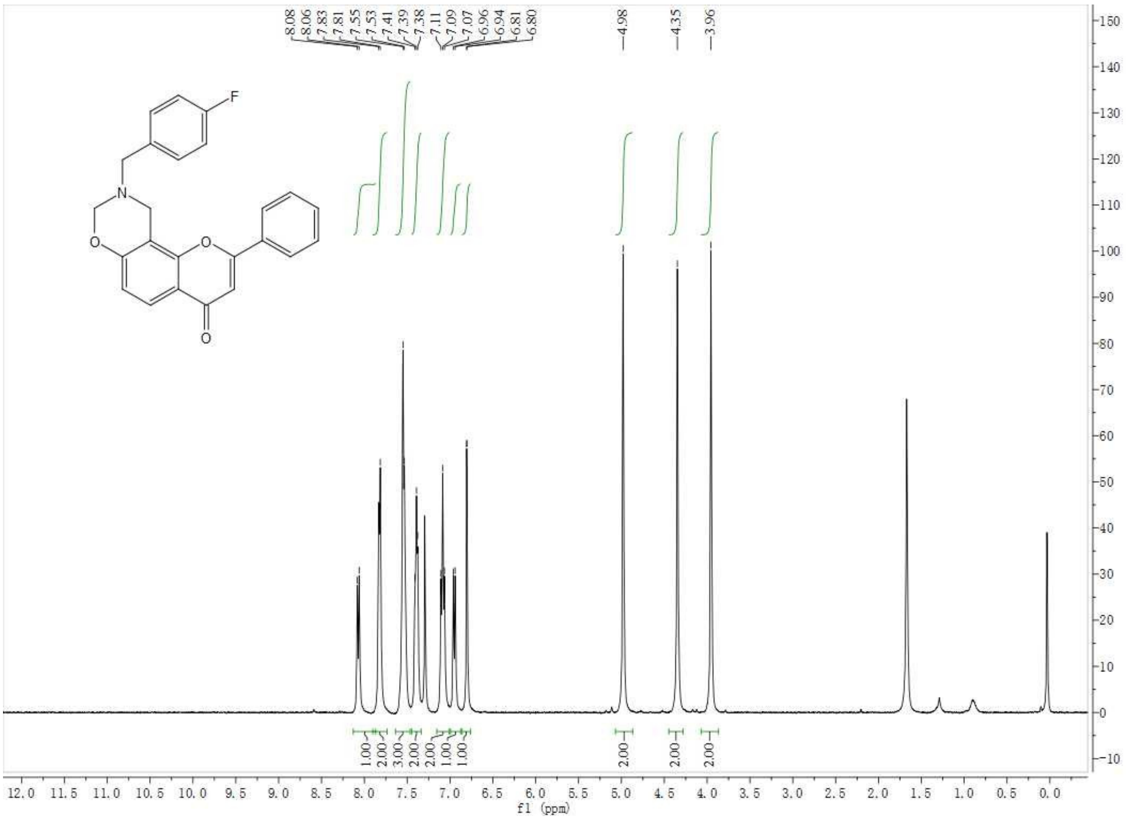

Figure S29. ^{1}H NMR spectrum of **6k**

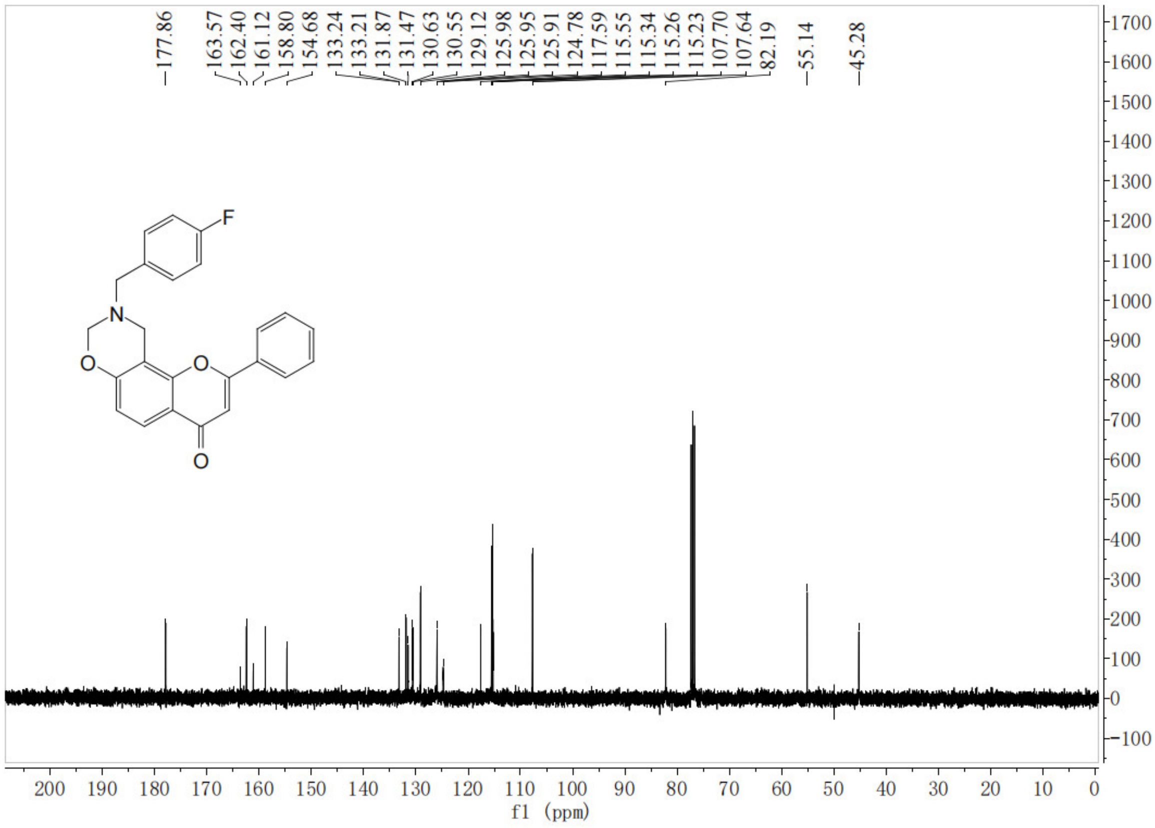

Figure S30. ^{13}C NMR spectrum of **6k**

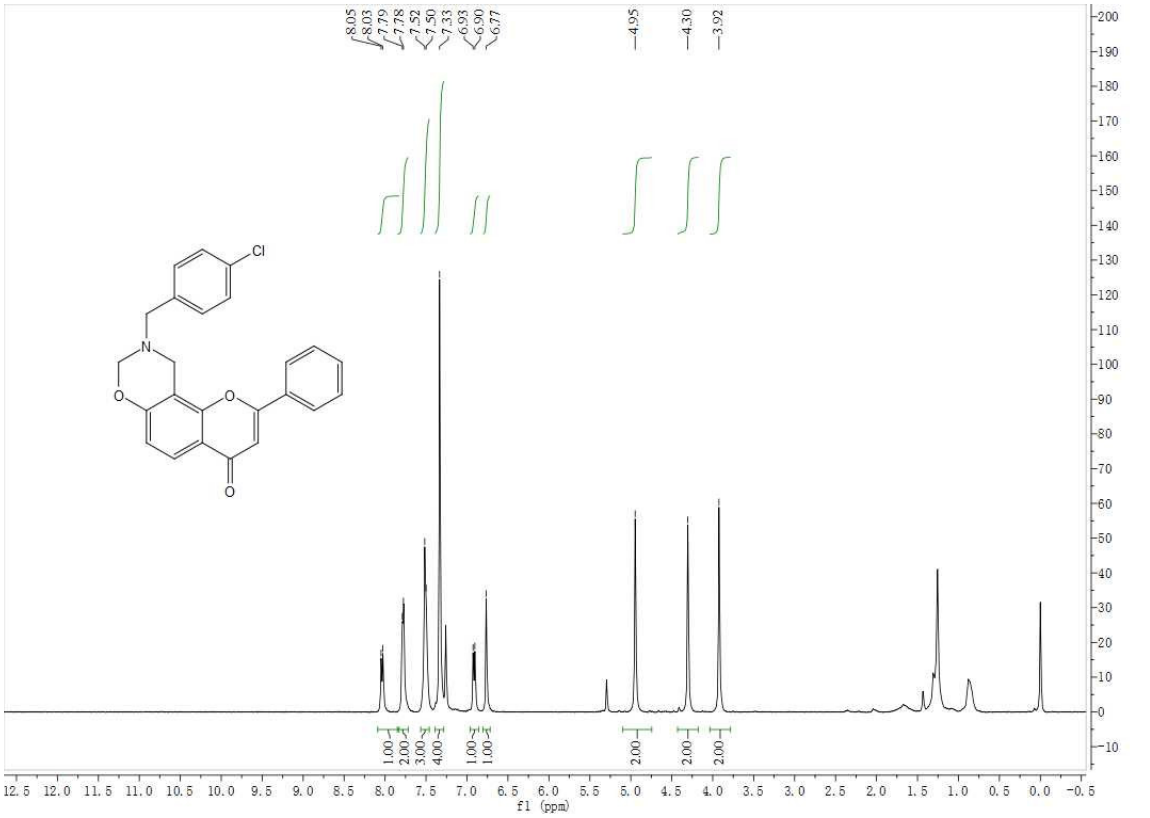

Figure S31. ^{1}H NMR spectrum of **6l**

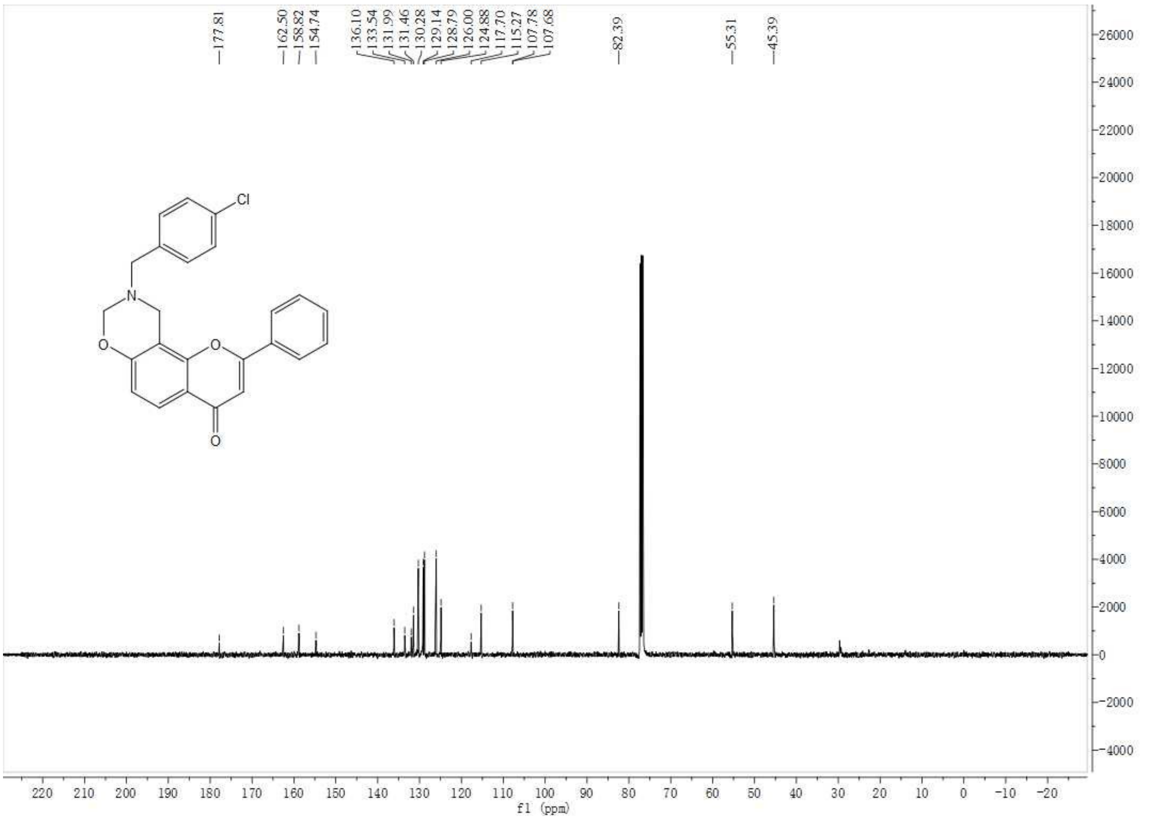

Figure S32. ^{13}C NMR spectrum of **6l**

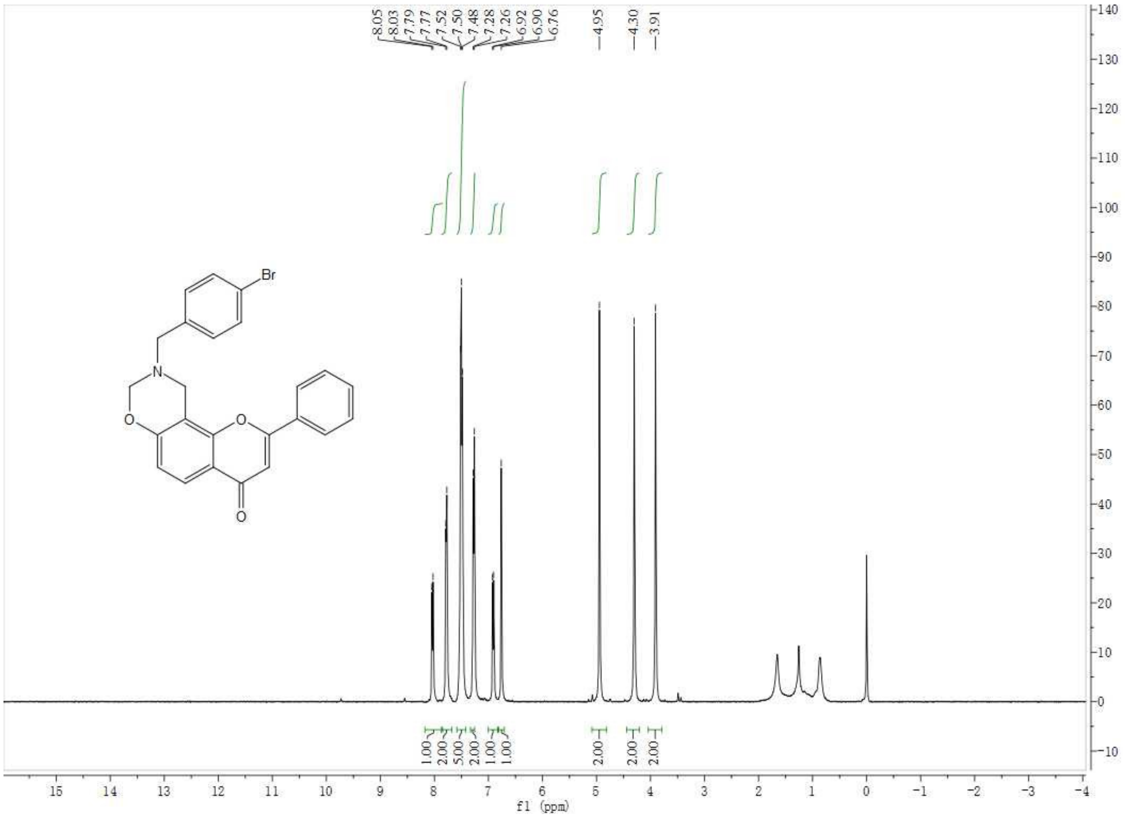

Figure S33. ^{1}H NMR spectrum of **6m**

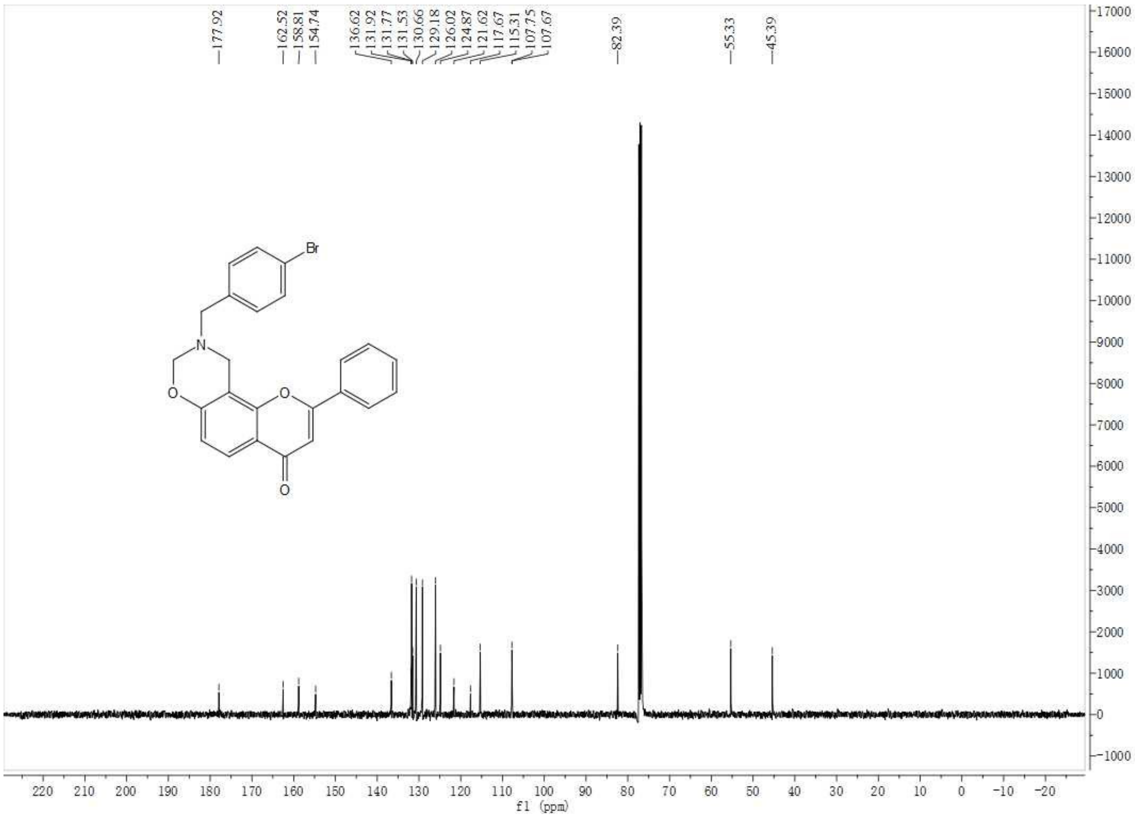

Figure S34. ^{13}C NMR spectrum of **6m**

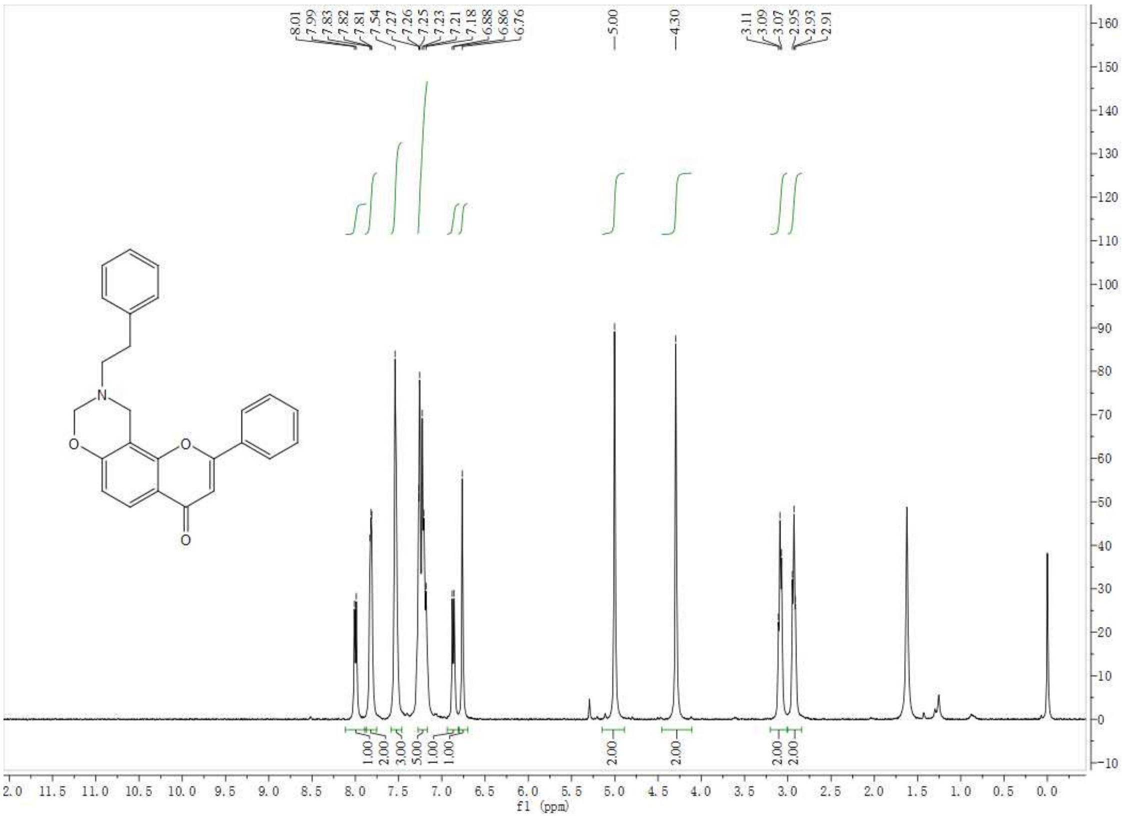

Figure S35. ^{1}H NMR spectrum of **6n**

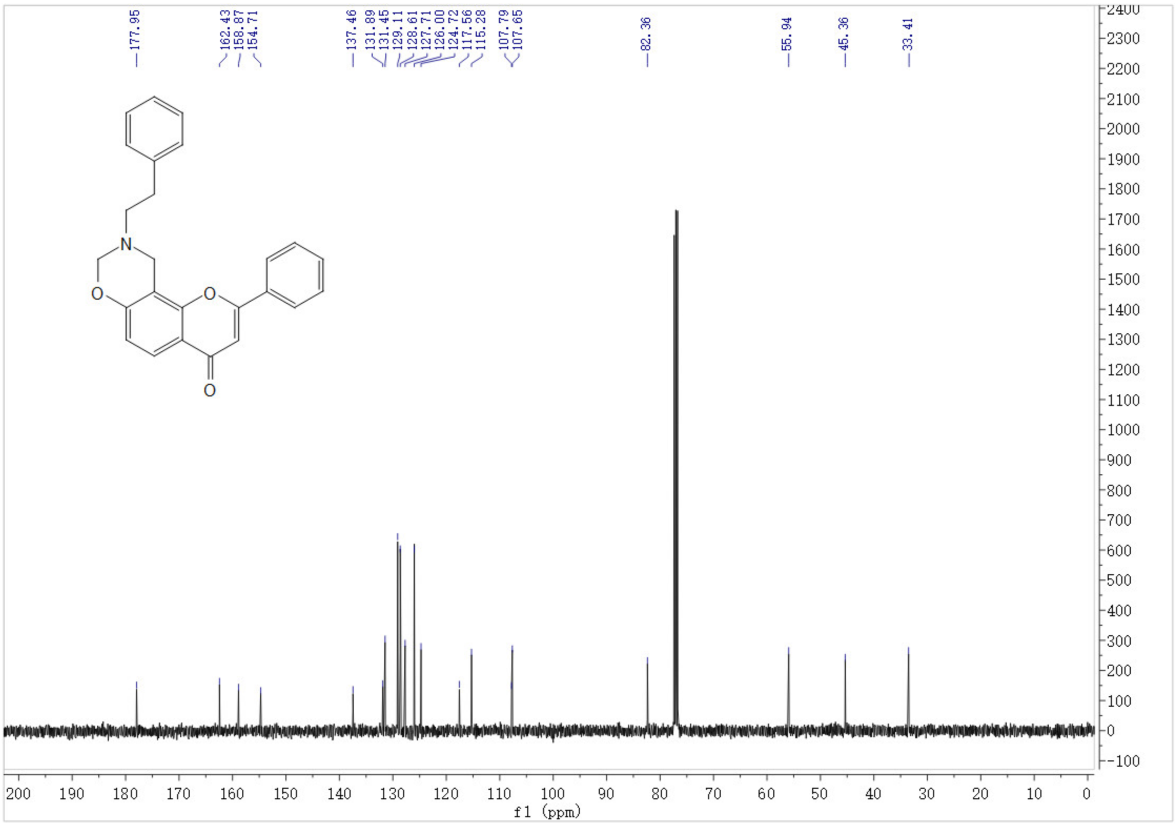

Figure S36. ^{13}C NMR spectrum of **6n**

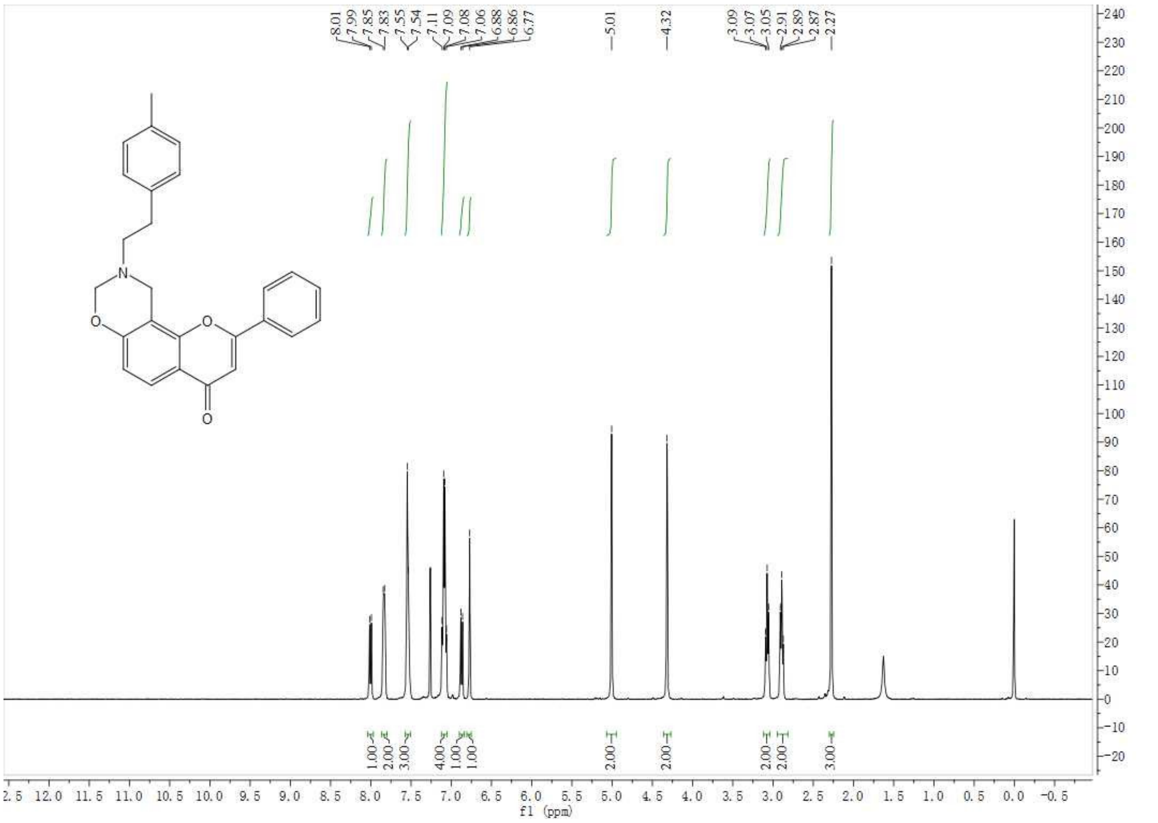

Figure S37. ^{1}H NMR spectrum of **6o**

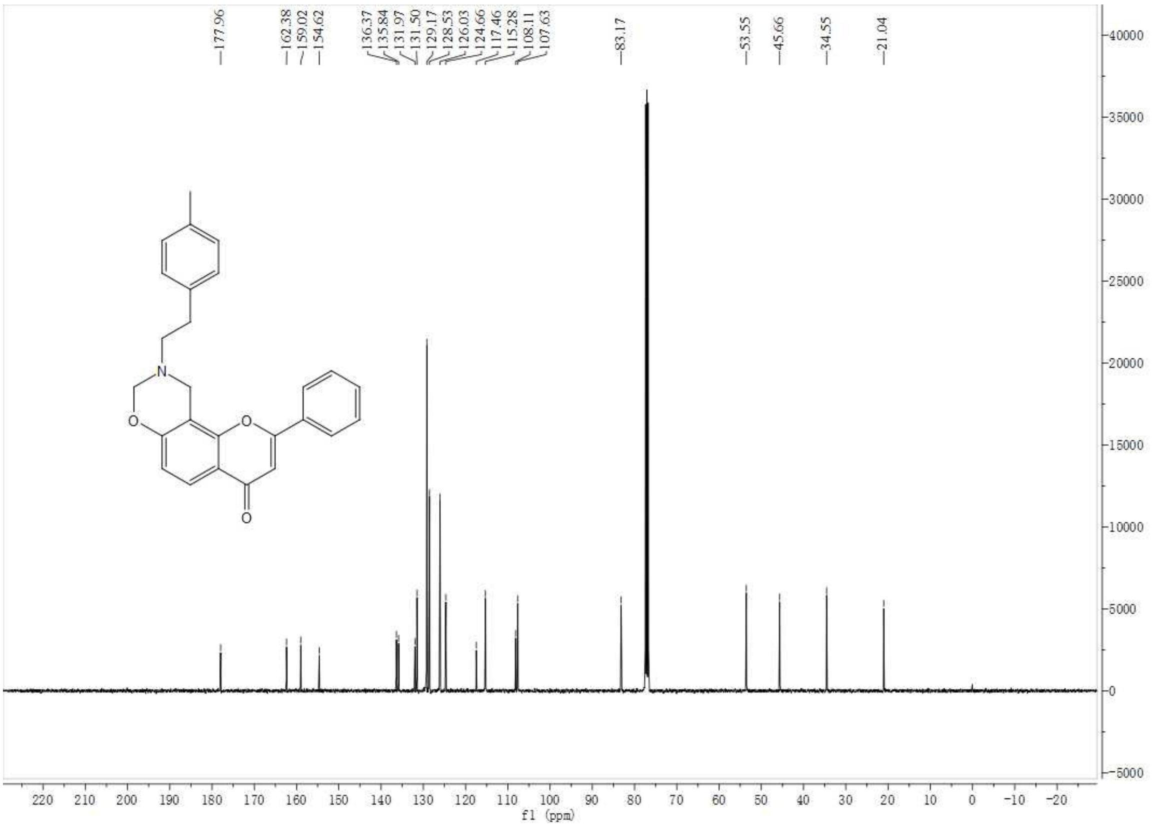

Figure S38. ^{13}C NMR spectrum of **6o**

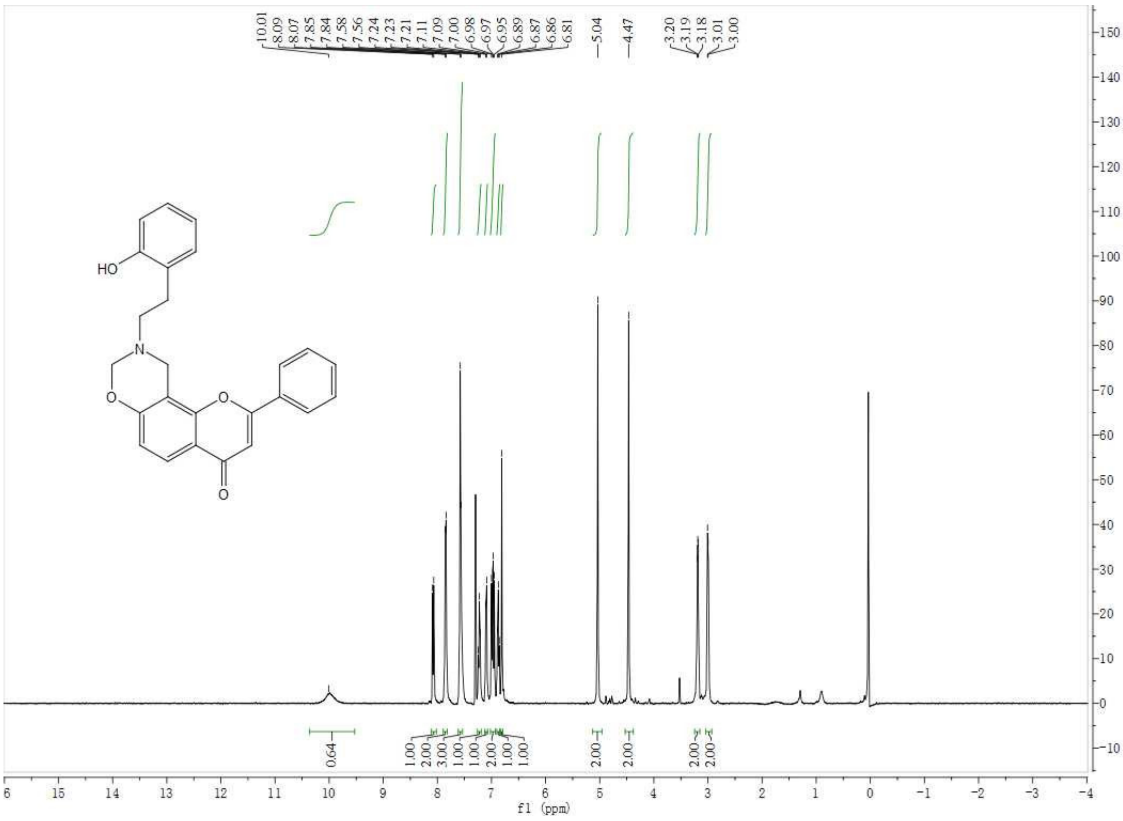

Figure S39. ^{1}H NMR spectrum of **6p**

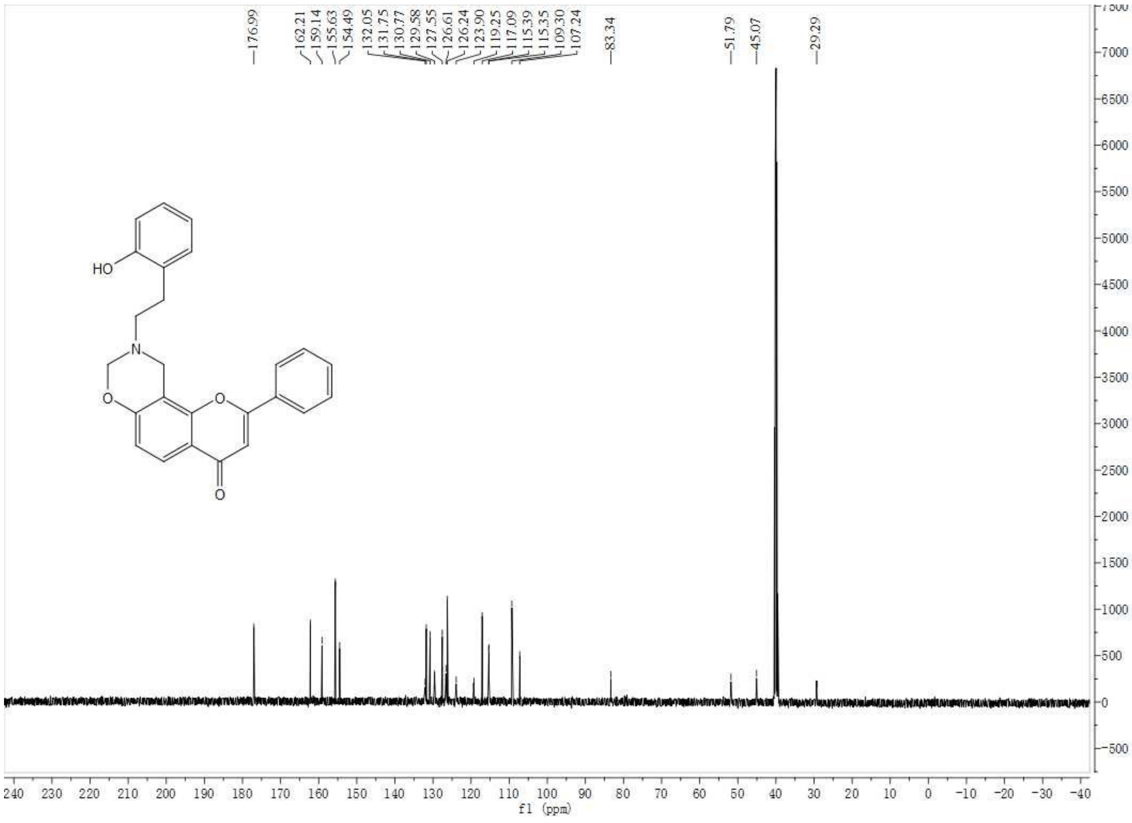

Figure S40. ^{13}C NMR spectrum of **6p**

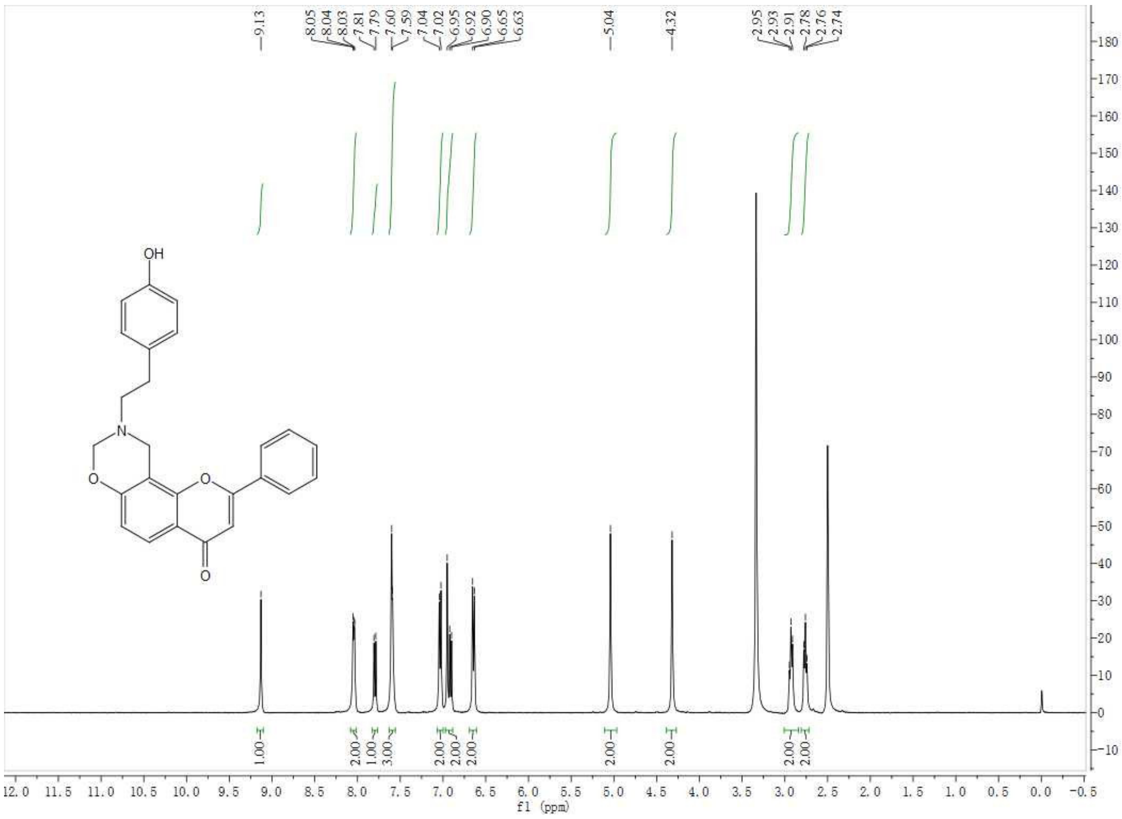

Figure S41. ^{1}H NMR spectrum of **6q**

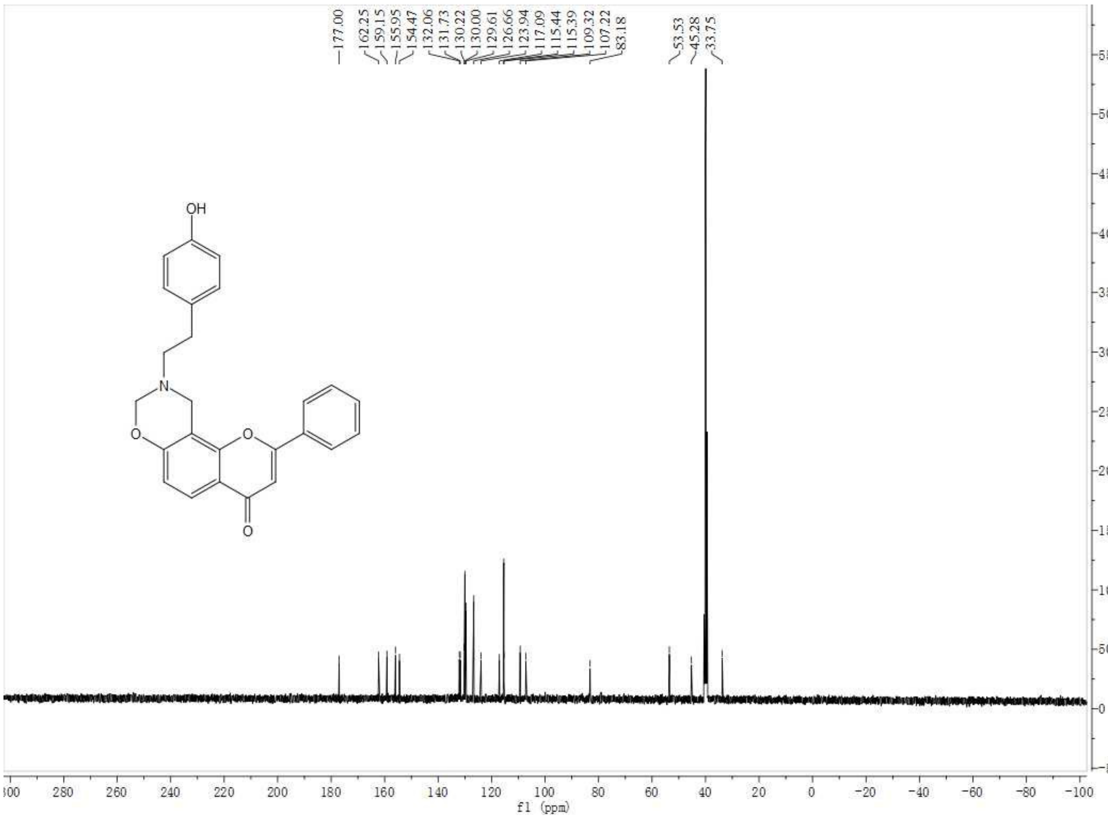

Figure S42. ^{13}C NMR spectrum of **6q**

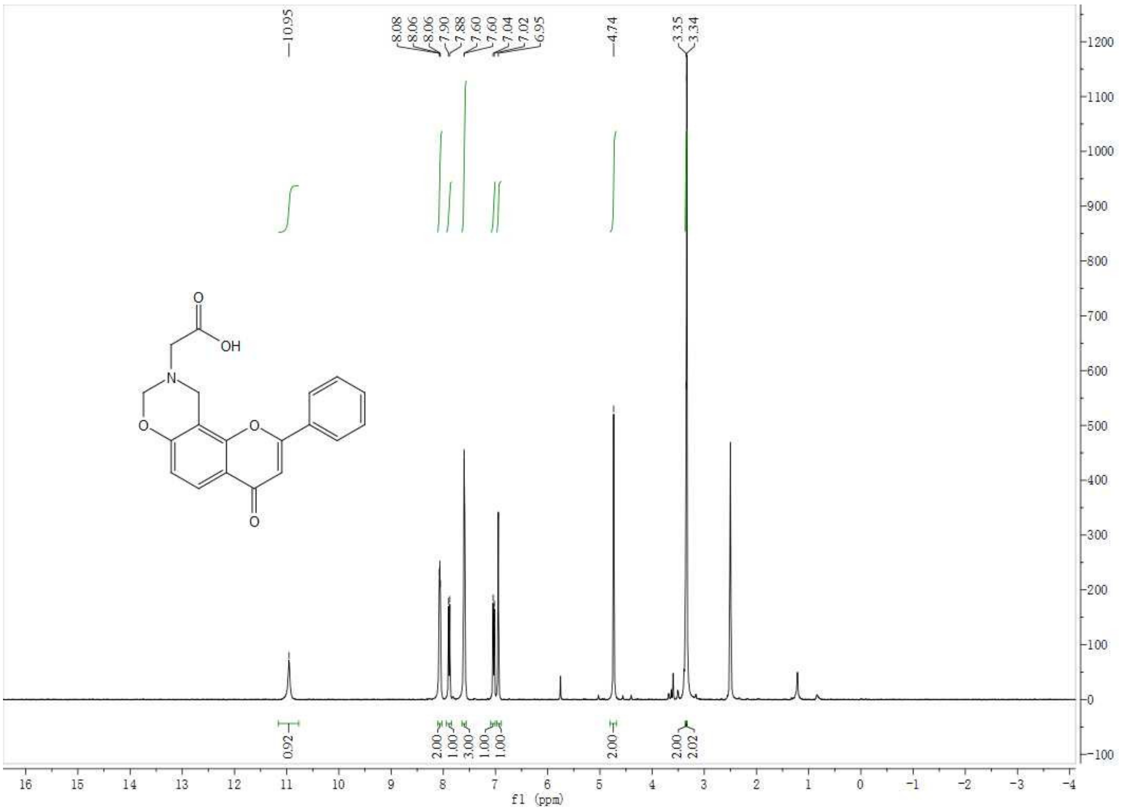

Figure S43. ^{1}H NMR spectrum of **6r**

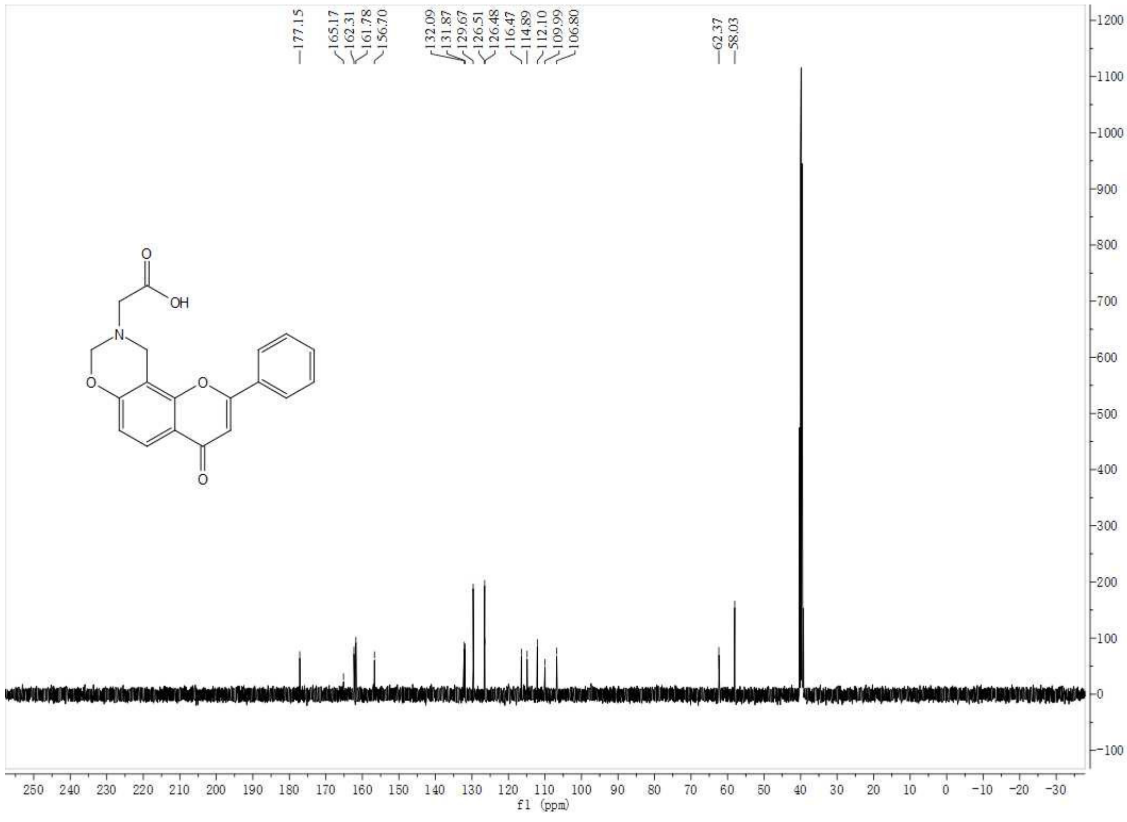

Figure S44. ^{13}C NMR ^{1}H NMR spectrum of **6r**
